# Effects of Organic Pollutants on Bacterial Communities Under Future Climate Change Scenarios

**DOI:** 10.3389/fmicb.2018.02926

**Published:** 2018-11-30

**Authors:** Juanjo Rodríguez, Christine M. J. Gallampois, Sari Timonen, Agneta Andersson, Hanna Sinkko, Peter Haglund, Åsa M. M. Berglund, Matyas Ripszam, Daniela Figueroa, Mats Tysklind, Owen Rowe

**Affiliations:** ^1^Department of Microbiology, University of Helsinki, Helsinki, Finland; ^2^Department of Chemistry, Umeå University, Umeå, Sweden; ^3^Department of Ecology and Environmental Sciences, Umeå University, Umeå, Sweden; ^4^Umeå Marine Research Centre (UMF), Umeå University, Hörnefors, Sweden; ^5^Department of Equine and Small Animal Medicine, University of Helsinki, Helsinki, Finland; ^6^MSCi ApS Laboratory, Skovlunde, Denmark; ^7^Helsinki Commission (HELCOM), Baltic Marine Environment Protection Commission, Helsinki, Finland

**Keywords:** bacterial community composition, organic pollutants, dissolved organic matter, climate change, Baltic Sea, metagenomics

## Abstract

Coastal ecosystems are highly dynamic and can be strongly influenced by climate change, anthropogenic activities (e.g., pollution), and a combination of the two pressures. As a result of climate change, the northern hemisphere is predicted to undergo an increased precipitation regime, leading in turn to higher terrestrial runoff and increased river inflow. This increased runoff will transfer terrestrial dissolved organic matter (tDOM) and anthropogenic contaminants to coastal waters. Such changes can directly influence the resident biology, particularly at the base of the food web, and can influence the partitioning of contaminants and thus their potential impact on the food web. Bacteria have been shown to respond to high tDOM concentration and organic pollutants loads, and could represent the entry of some pollutants into coastal food webs. We carried out a mesocosm experiment to determine the effects of: (1) increased tDOM concentration, (2) organic pollutant exposure, and (3) the combined effect of these two factors, on pelagic bacterial communities. This study showed significant responses in bacterial community composition under the three environmental perturbations tested. The addition of tDOM increased bacterial activity and diversity, while the addition of organic pollutants led to an overall reduction of these parameters, particularly under concurrent elevated tDOM concentration. Furthermore, we identified 33 bacterial taxa contributing to the significant differences observed in community composition, as well as 35 bacterial taxa which responded differently to extended exposure to organic pollutants. These findings point to the potential impact of organic pollutants under future climate change conditions on the basal coastal ecosystem, as well as to the potential utility of natural bacterial communities as efficient indicators of environmental disturbance.

## Introduction

Marine coastal environments are especially vulnerable to change due to the dual influence of the surrounding terrestrial ecosystems and the connecting water-bodies. Coastal systems are thus highly dynamic and can be strongly influenced by climate change (Stocker et al., 2013), anthropogenic activities (e.g., pollution), and a combination of the two pressures (Robinson et al., 2004). Pollutants can have various effects on marine ecosystems, such as direct toxicity, persistent negative effects, or bioaccumulation (Kallenborn et al., 2012; Andersson et al., 2015). Furthermore, the physicochemical environment can alter the behavior of these substances (Ripszam et al., 2015a,b). Moreover, the northern hemisphere is predicted to undergo an increased precipitation regime, leading in turn to higher terrestrial runoff and increased river inflow (up to 15–22%) to the Baltic Sea by the end of the century (Meier et al., 2011). This increased runoff will transfer terrestrial dissolved organic matter (tDOM) to coastal waters, increasing the concentration of substances such as: dissolved organic carbon (DOC), humic substances, and nutrients (e.g., nitrogen and phosphorus); thereby altering the physicochemical environment (Figueroa et al., 2016). Such changes can directly influence the resident biology, particularly at the base of the food web and can influence the partitioning of contaminants (Cole et al., 1988; Ripszam et al., 2015a).

The effects of higher tDOM inputs can be observed in the different basins of the Baltic Sea, where an increasing gradient in tDOM concentration is found from South to North (Sandberg et al., 2004; Rowe et al., 2018). The southern regions receive terrestrial loadings richer in nutrients (particularly phosphorus), while the northern regions are exposed to terrestrial loadings richer in humic substances (Andersson et al., 2015). In addition, the higher concentrations of humic substances and colored dissolved organic matter (CDOM) typically found in the northern areas of the Baltic Sea (Harvey et al., 2015; Paczkowska et al., 2016) have been observed to increase light attenuation, which can have negative consequences for phytoplankton production and biomass (Roulet and Moore, 2006; Frigstad et al., 2013) and alter the bacterial metabolic response, as well as the characteristics of the processed DOM (Berggren and del Giorgio, 2015; Rowe et al., 2018). Allochthonous DOM (ADOM) has been implicated in the uncoupling of phytoplankton and bacterioplankton production due to the presence of an external carbon source available for bacteria (Andersson et al., 2013; Figueroa et al., 2016). As a consequence, the northern regions of the Baltic Sea show a trophic balance tipped toward net heterotrophy (Figueroa et al., 2016), which has the potential to decrease pelagic food web efficiency due to increased losses of energy and carbon during transfer through more complex food webs (Sommer et al., 2002; Berglund et al., 2007). Furthermore, the quantitative and qualitative composition of ADOM can influence bacterial production (BP) and also lead to differences in bacterial community composition and functioning (Teira et al., 2009; Grubisic et al., 2012), factors of major potential significance under future predicted climate conditions (Lindh et al., 2015; Traving et al., 2017).

The fate and redistribution of organic pollutants are also of major concern in regard to projected climate change impacts on marine environments (EU Directive 2008/105/EC; Kallenborn et al., 2012; Ripszam et al., 2015a). The Baltic Sea is subjected to inputs of organic pollutants (persistent and emerging) from diverse anthropogenic sources, such as industrial processes or agricultural products (Helcom, 2009, 2010), which are channeled into marine environments via direct urban and industrial effluents, terrestrial runoff, ocean currents, or atmospheric transport and deposition (Fernández and Grimalt, 2003; Htap, 2010; Bidleman et al., 2015). Weather factors linked to climate change, such as temperature, wind speed, precipitation patterns, ice cover, and rising sea levels, are projected to increase the mobilization of organic pollutants from their source to the marine environment (Schiedek et al., 2007; Lamon et al., 2009; Kallenborn et al., 2012). In addition, quality and quantity of DOC have been shown to play an important role in the fate of organic pollutants in the Baltic Sea and other brackish water environments due to differential sorption of organic pollutants to DOC. Such factors influence the solubility, transport, bioavailability, and food web transfer of these organic pollutants (Krop et al., 2001; Roulet and Moore, 2006; Ripszam et al., 2015a). It is thus expected that they will have a major impact on the Baltic Sea marine environment under predicted future climate change scenarios. While some studies have explored the consumption and metabolism of organic pollutants by different bacterial taxa or their potential for bioremediation (Tian et al., 2008; Dash et al., 2013; Nzila, 2013; Kang, 2014), little is known about effects of organic pollutants on the structure of naturally occurring marine bacterial communities, particularly under predicted future climate change conditions.

In order to determine the effects of increased tDOM concentration, occurrence of organic pollutants (sub-acute toxicity levels), and the combined effect of these two factors on pelagic bacterial communities, a mesocosm study was carried out over 5 weeks employing natural sea water from a coastal area of the Bothnian Sea. Bacterial communities in the mesocosms were sampled once per week, and characterized using high-depth sequencing of the 16S rRNA gene fragment. The impact of the mesocosm treatments on bacterial community structure, as well as on individual bacterial operational taxonomic units (OTUs), were analyzed with multivariate statistical techniques.

## Materials and Methods

### Experimental Design

The mesocosm experiment was performed in May–July 2013 over a 6-week period at the Umeå Marine Sciences Center (UMF), Sweden. Twenty-four polypropylene tanks (1 m^3^) were filled with 300-μm filtered water from the northern coastal Baltic Sea (63°33′27.0″N 19°50′7.5″E) (Supplementary Figure S1), and thus included a natural seawater community of bacteria, phytoplankton, protozoa, and mesozooplankton. Furthermore, 10 larval perch individuals were added to each mesocosm as top consumer. Two variables were altered in a 2 × 2 factorial design: DOC concentration and pollutant addition; and identical mesocosm treatments were exposed to two temperatures (15 and 18°C), as described in detail by Ripszam et al. (2015b). However, temperature treatment was excluded from the study to focus on the role of pollutants and tDOM, both parameters considered to be major climate change-related issues in this region (Andersson et al., 2015). The 3°C change was considered to likely influence functional and metabolic aspects more strongly than community aspects over the relatively short experimental duration. Thus, only samples from the 15°C treatment were analyzed in this present study. Overall, the treatments in this study were (1) control (CONT) – ambient water conditions (i.e., natural DOC concentrations and no pollutants added), (2) soil extract addition (SOIL-EX) – elevated DOC concentrations by addition of extracted terrestrial matter, (3) pollutant addition (POLL) – addition of a pollutant mixture, and (4) pollutant and soil extract addition (SOIL–POLL) – addition of extracted terrestrial matter and the addition of pollutants. All treatments were applied in triplicate, making a total of 12 mesocosms.

### Treatment Additions

SOIL treatment – DOC increase was driven by the addition of a tDOM extract formed from soil material collected locally on the banks of the Öre river (Lefébure et al., 2013). In brief, this process involved collected soil being mixed with Milli-Q water and ion exchange resin (Amberlite IRC 7481) for 48 h at 4°C, prior to filtering through a 90-μm mesh. Carbon, phosphorous, and nitrogen concentrations were measured to enable calculation of volumes to be added. The addition of soil extract was done to increase the ambient DOC concentration (ca. 4 mg L^-1^) to 6 mg L^-1^, a 50% increase from current DOC levels, as predicted in coastal climate change scenarios for the region (Eriksson Haägg et al., 2010).

POLL treatment – pollutants were selected from a list of hazardous substances presented by the European Commission, 2008 (EU Directive 2008/105/EC). This list includes different groups of organic pollutants with clearly differentiated structural elements (e.g., anilines and phenols with different degrees of bromination), as well as polar and non-polar properties. The selected chemicals represent compounds commonly isolated in the Baltic Sea, including: (1) persistent pollutants [polycyclic aromatic hydrocarbons (PAHs); polychlorinated benzenes (PCBz); polychlorinated biphenyls (PCBs); and organochlorine pesticides (OCPs)], which have been described to be very bioaccumulative and have hazardous effects on living organisms (WHO, 2000a,b); and (2) emerging pollutants, such as anthropogenic organophosphates (used as plasticizers and flame retardants) and biogenic low molecular weight brominated compounds, which have been shown to have carcinogenic and neurotoxic effects (European Commission, 2012; van der Veen and de Boer, 2012, EU Directive 2012/125/EC). The selected pollutants fell into nine categories according to their chemical composition (Supplementary Figure S2). A detailed list of those compounds constituting the pollutant mixture, the analytical methods used, their concentrations, and trends during the experiment is described in Ripszam et al. (2015b).

### Experimental Time Plan and Sampling

**FIGURE 1:**
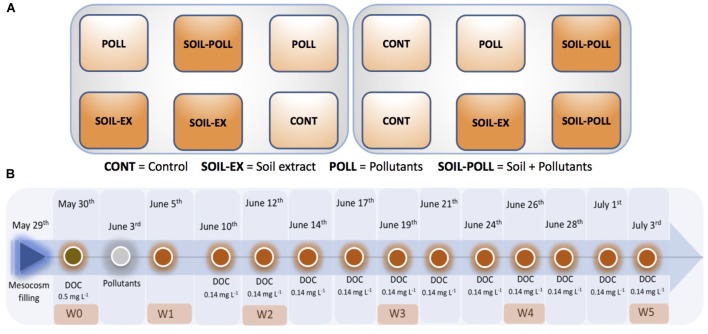
Layout of the experimental treatments **(A)** and experimental timeline **(B)**. At every timepoint showed **(B)**, 20 L of water was collected for sampling and subsequently replaced by adding 20 L of fresh seawater filtered through 1 urn W0–W5 refer to the sampling points at which samples for bacterial community analyses were collected. A soil extract (tDOM) was sequentially added throughout the experimental time to increase the ambient DOC concentration from ca. 4 to 6 mg L^-1^. Organic pollutants were added as a cocktail containing a mixture of different pollutants at sub-acute toxicity levels (0.1 μg L^-1^).

On the 29 May, the mesocosms were filled with 946 L of seawater (salinity ∼3) collected from the research stations central supply of seawater, whose intake is placed 1 km offshore of the UMF facility (63°33′27.0″N 19°50′7.5″E). A soil extract, representative of a quarter of the total planned additions (0.5 mg L^-1^ of the 2 mg L^-1^ addition described above), was then added to the respective treatment tanks (Figure 1) after approximately 1 day of food web stabilization had been allowed. The final 75% increase in DOC concentration was achieved by sequential smaller additions (0.14 mg C L^-1^, 6.8% of total planned additions) of soil extract (tDOM) added every third day over the duration of the experiment. Following the first tDOM addition, the mesocosms were left to equilibrate for approximately 4 days, after which a cocktail of pollutants was added at sub-acute toxicity levels (0.1 μg L^-1^) to the respective treatment tanks (Figure 1). According to Stepanauskas et al. (2002), ∼30% of the dissolved organic nitrogen (DON) and ∼75% of dissolved organic phosphorus (DOP) are potentially bioavailable for bacterioplankton in the Baltic Sea. Therefore, those mesocosms without soil addition received the corresponding DIN and DIP supplements (weekly) in order to match the concentrations in the mesocosms with soil addition and to balance for bioavailability. Twenty liters of water (inclusive of sampling waters) was removed from each tank three times a week throughout the experiment and replaced with fresh seawater that had been passed through a 1-μm filter. Finer filtration or the re-fill water was hampered due to technical constrains during the experiment. The mesocosms were sampled weekly and a number of physicochemical and biological parameters were measured on each occasion and in every mesocosm tank, as described below.

Photosynthetically active radiation (PAR) was measured using a PAR Licor sensor (LICOR –193SA). The measurements were performed weekly in each mesocosms at five special positions at the surface (45 and 90 cm depths). The resulting 15 data points were used to calculate the average PAR values for each mesocosm.

### Carbon and Nutrient Analysis

Total phosphorous (Ptot), total nitrogen (Ntot), dissolved inorganic phosphorous (DIP), and dissolved inorganic nitrogen (DIN) were determined using a 4-channel auto-analyzer (Quaatro Marine, Bran & Luebbe^®^) according to the Swedish Standards Institute and HELCOM (Grasshoff et al., 1983). Duplicate 12 mL DOC samples were filtered through a 0.2-μm filter (Whatman^®^GF/F) according to Norrman (1993). These samples were then acidified with 375 μL of 1.2 M HCl and stored at -20°C before analysis. DOC was measured by the high-temperature catalytic oxidation method using a Shimadzu TOC-5000 instrument (Shimadzu Corporation) with platinum-coated Al_2_O_3_ granulates as a catalyst.

### Phytoplankton Parameters

Primary production (PP) was measured using the ^14^C method (Gargas, 1975). Five milliliter samples of seawater were added to four 20 mL transparent polycarbonate tubes and mixed with 7.2 μL Na_2_^14^CO_3_ (Centralen Denmark, specific activity = 100 μCi mL^-1^). The tubes were incubated *in situ* for approximately 3 h at 1 m depth, with one of the replicates being incubated in dark. Next, a sub-sample of 5 mL from each tube was mixed with 150 μL 6 M HCl. After 30 min of bubbling, 15 mL scintillation cocktail was added and thoroughly mixed. The samples were analyzed in a scintillation counter (Beckman Coulter LS 6500/Packard Tri-Carb 1600 TR) and net PP was calculated as describe in Andersson et al. (1996).

Samples for chlorophyll-*a* (Chl-*a*) were filtered onto Whatman^®^GF/F filters, extracted overnight in the dark in 95% ethanol, and then measured on a Perkin Elmer LS 30 spectrofluorometer (Waltham^®^, Middlesex, MA, United States) operating at excitation and emission wavelengths of 433 and 673 nm, respectively.

### Bacterial Production and Abundance

Bacterial net production (BP) was measured using the [^3^H-methyl]-thymidine incorporation method (Fuhrman and Azam, 1982). Four 1 mL replicates and one killed control were incubated in the dark at 15°C for 1 h with [^3^H-methyl]-thymidine (84 Ci mmol^-1^, Perkin Elmer^®^, Wellesley, MA, United States) at a final concentration of 24 nM. The incubation was stopped by the addition of 100 μL of ice-cold 50% trichloroacetic acid (TCA) and the samples were then centrifuged in the cold at 13,000 rpm for 10 min. The resulting pellet was washed with 5% TCA and, and after adding 1 mL of scintillation cocktail the samples were analyzed in a scintillation counter (Beckman Coulter LS 6500/Packard Tri-Carb 1600 TR). BP was calculated using a conversion factor of 1.4 × 1018 cells mol^-1^ (Wikner and Hagström, 1999) and a carbon conversion factor of 20 fg C cell^-1^ (Lee and Fuhrman, 1987).

Bacterial abundance (BA) was measured using a BD FACSVerseTM flow cytometer (BD Biosciences) fitted with a 488-nm laser (20 mW output). The samples were stained with SYBR Green I (Invitrogen) to a final concentration of 1:10,000 (Marie et al., 2005) and diluted with 0.2 μm of filtered seawater. They were run at a low flow rate of 30 μL min^-1^ with an acquisition time of 2 min. As an internal standard, 1 μm microspheres (Fluoresbrite plain YG, Polysciences) were added to each sample (∼10^6^mL^-1^). Forward light scatter (FSC), side light scatter (SSC), and green fluorescence from SYBR Green I (527 ± 15) were measured to estimate bacteria abundance. Bacterial biomass was then calculated using a conversion factor of 20 fg C cell^-1^ (Lee and Fuhrman, 1987). Doubling time was calculated considering BA (Velimirov and Walenta-Simon, 1993), and the timeframe comprised between week 1 and week 2 (i.e., highest growth rate recorded over the experiment) was utilized.

### Total Community DNA Extraction and PCR Amplification

Samples for DNA extraction consisted of 500 mL of seawater filtered onto 0.22 μm cellulose–acetate filters (Gelman Supor^®^). Each replicated mesocosm was sampled once weekly (except for week 4) and the samples were stored in 1 mL Tris–EDTA buffer (VWR, Radnor, PA, United States) at -80°C. DNA was extracted using an E.Z.N.A.^®^Soil DNA Kit (Omega Bio-Tek, United States) according to the manufacturer’s protocol. The V3–V4 regions of the bacterial 16S rRNA gene were amplified using the primers 341F (5′-CCTACGGGNGGCWGCAG-3′) and 805R (5′-GACTACHVGGGTATCTAATCC-3′) (Eurofins, Germany). PCR conditions were 95°C for 2 min, followed by 25 cycles at 95°C for 30 s, 55°C for 30 s, 72°C for 45 s, and a final extension at 72°C for 10 min. Dual-indexed DNA barcodes (Eurofins, Germany) were employed for multiplexing the amplicon libraries in a second PCR reaction using the same PCR conditions. Duplicate PCR reactions were performed in 25 μL volumes containing 1–10 ng DNA template, 12.5 μL 2× Phusion Mastermix (ThermoScientific), 1.25 μL of each adapter (at 10 pmol), and nuclease-free water to complete the final total volume. Duplicate PCR products were pooled, cleaned, and purified using AMPure XP beads (Beckman Coulter, Inc., Brea, CA, United States) and DNA was quantified using a Qubit^®^dsDNA BR Assay Kit (Thermo Scientific Fisher, United States).

### 16S rRNA Gene Sequencing and Bioinformatics Analysis

Polymerase chain reaction products from each mesocosm sample were pooled at equimolar concentration and submitted for 2 × 300 paired-end sequencing on an Illumina^®^MiSeq v3 platform at the Biomedicum Functional Genomics Unit (FuGU), Helsinki (Finland). The FASTQ files generated by Illumina MiSeq were processed in Mothur v. 1.40.5 (Schloss et al., 2009) according to the Mothur MiSeq standard operating procedure (SOP), with the alterations described in Timonen et al. (2016). The trimming step was implemented by executing an additional screen.seqs command to remove those contigs presenting a length of the overlapping region from forward and reverse reads below 25 bp. A further screen.seqs command removed ambiguous sequences and homopolymers. The trimmed and denoized sequences were aligned against SILVA reference database (release 119) (Quast et al., 2012; Yilmaz et al., 2013) and taxonomically classified with a bootstrap confidence threshold of 80%. As implemented by Mothur 1.40.5, chimeric sequences were checked and removed using the VSEARCH algorithm (Rognes et al., 2016). The resulting 16S rRNA gene sequences were clustered into OTUs with at least 97% similarity. Sequences have been deposited with links to BioProject accession number PRJNA435425 in the NCBI BioProject database^1^.

### Statistical Analysis

All statistical analyses were performed in the *R* project environment (v. 3.3.0). To investigate whether the physico-chemical parameters, BP, and abundance, as well as phytoplankton parameters, were significantly different between treatments and over time, two-way repeated measurement ANOVA was performed at 95% family-wise confidence level.

The *phyloseq* package (v. 1.19.1) was used to calculate relative abundance at different taxonomic levels (i.e., class and genus). For all other statistical analyses, the raw counts were rarefied by randomly down-sampling to the smallest library size (59,752 reads). OTUs including less than five reads in the entire data set were excluded for further analyses. Principal coordinate analysis (PCoA) was applied to visualize variance in the structure of the bacterial communities based on pairwise Bray–Curtis dissimilarities between samples (function *cmdscale*, *vegan* package, v. 2.4.3). A generalized linear model (GLM) (Warton et al., 2012) was applied to identify the bacterial taxa significantly contributing to the differences in bacterial community composition. The GLM was carried out in pairwise comparisons between the treatments and over time (sampling occasions) at both class and genus levels using the *mvabund* package (v. 3.12.3). The *p*-values were adjusted using a resampling-based implementation of Holm’s step-down multiple testing procedure, as implemented in Wang et al. (2012). The likelihood ratio (LR) test statistics was employed to test the hypotheses of no treatment and no temporal effects. In addition, Tukey’s Honest Significant Differences tests were applied on the OTUs found at week 5 to identify significant differences in OTU composition between the different treatments. In order to focus this approach, OTUs containing less than 100 reads in total (entire data set) were discarded for this analysis. Pearson’s correlation analyses were carried out using the function *rcorr* in *Hmisc* package (v. 4.0.3) to investigate possible linear dependence between concentration of pollutants and abundance of relevant OTUs from GLM test, as well as between the different environmental factors.

Alpha diversity from normalized 16S rRNA gene sequences was calculated based on Shannon’s diversity index using the function *estimate_richness* in the *phyloseq* package (v. 1.19.1). Significant differences in the Shannon’s index were tested using two-way repeated measurement ANOVA and Tukey’s honest significant differences tests with pairwise comparisons.

## Results

### Organism Dynamics

**FIGURE 2:**
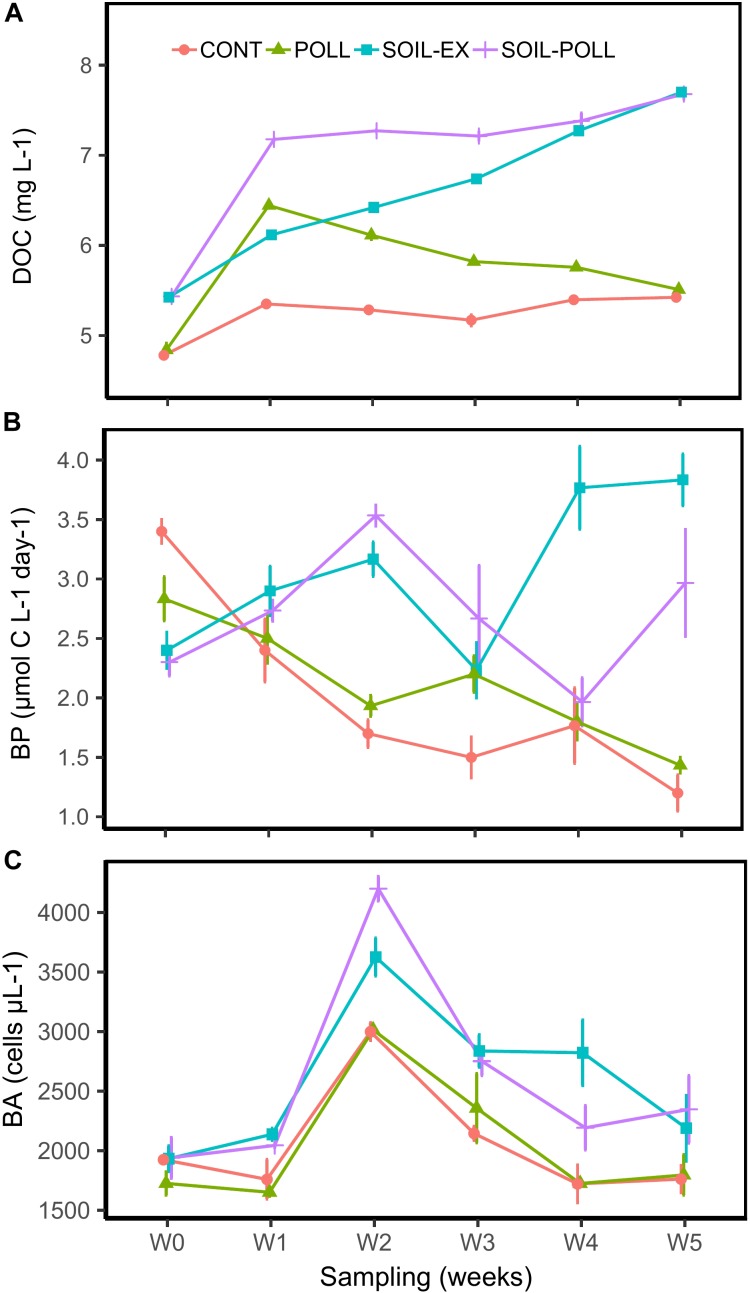
Development of DOC concentration **(A)**, bacterial production **(B)**, and BA **(C)** throughout the experiment from the different treatments. CONT, control; POLL, pollutants addition; SOIL-EX, DOC addition; SOIL–POLL, DOC + pollutants addition. Error bars represent the standard deviation (*n* = 3).

The experiment started at the peak of the spring bloom. PP was thus highest at the beginning of the experiment and decreased as inorganic nutrients were consumed (Supplementary Figures S3D,F). In the early part of the experiment, fish heavily grazed on zooplankton (by circa week 2), reducing zooplankton biomass circa 10-fold by the end of the experiment. This process released ciliates and other potential bacterial grazers from predation (provisional assessment, data not shown). Heterotrophic bacteria showed an overall increase from the start to the middle of the experiment (Figure 2C), where after they decreased in abundance.

### Pollutants

The concentration of pollutants in the water strongly declined during the initial days directly after their addition, and remained at low levels throughout the experiment (Supplementary Figure S2). A rapid redistribution of these compounds took place with pollutants moving from the aqueous phase to DOC, particles, biota, the walls of the containers, and via evaporation. The partitioning of pollutants to DOC, biota, and particles was particularly pronounced under higher DOC conditions (SOIL–POLL treatment). Sedimentation was also higher in SOIL–POLL treatment, leading to higher pollutant losses in this treatment compared to POLL treatment. A detailed description of the pollutants and their behavior can be found in Ripszam et al. (2015b).

### DOC and Physicochemical Parameters

Dissolved organic carbon concentration was significantly higher in the treatments supplied with soil extract (SOIL-EX and SOIL–POLL) compared to the treatments with no addition (CONT and POLL) (*p* < 0.0001, $\eta_{p}^{2}$ = 0.94) (Figure 2A). DOC accumulated in the system increasing in concentrations by ∼30% over the duration of the experiment. The resulting end point (week 5) differences between DOC and non-DOC treatments were also in the range of 30% (Figure 2A and Supplementary Table S1). Addition of pollutants resulted in an additional increase in DOC concentrations followed by a steady decrease over the duration of the experiment, re-converging with the respective non-pollutant treatments by week 5 (Figure 2A and Supplementary Table S1). This increase in DOC concentration was likely due to the pollutants themselves and the use of methanol as the solvent for the cocktail of pollutants (as described in Ripszam et al., 2015b), which alone contributed 0.79 mg/L of carbon to those mesocosm tanks.

The addition of soil extract also led to an increase in Ptot and Ntot concentrations (Supplementary Figures S3C,E), showing significant differences between tDOM and non-tDOM treatments (Ptot, *p* < 0.0001, $\eta_{p}^{2}$ = 0.86; Ntot, *p* < 0.0001, $\eta_{p}^{2}$ = 0.96). In non-tDOM treatments, the concentration of both Ptot and Ntot gradually decreased toward the end of the experiment. Although Ptot slightly decreased in all treatments until week 2, its concentration subsequently increased toward week 5 in the tDOM treatments. Similarly, DIP markedly declined during the first 2 weeks in all treatments and then increased toward the end of the experiment only in tDOM treatments. This increase was particularly pronounced in SOIL-EX, though somewhat deferred and lesser in the SOIL–POLL, and in non-tDOM treatments DIP generally remained below the detection limit (Supplementary Figure S3D). On the other hand, DIN concentrations generally showed a decreasing trend over the duration of the experiment (Supplementary Figure S3F).

The addition of soil extract also resulted in the attenuation of the light intensity (PAR) entering the water column. Thus, while the control mesocosms showed an average light intensity of ∼250 μmol PAR quanta m^-2^ s^-1^, the tDOM mesocosms showed on average ∼150 μmol PAR quanta m^-2^ s^-1^, which constitutes a decrease in light intensity by ∼60%.

### Bacterial Production, Bacterial Abundance, and Association With Other Variables

In general, BP was significantly higher in the tDOM treatments than in non-tDOM treatments (*p* < 0.0001, $\eta_{p}^{2}$ = 0.65) (Figure 2B). In the non-tDOM treatments, BP decreased over the course of the experiment, whereas in the tDOM treatments the trend was the opposite. BA followed a similar pattern in all treatments (Figure 2C), peaking in week 2. However, tDOM treatments showed significantly higher BA (*p* < 0.0001, $\eta_{p}^{2}$ = 0.60) than non-tDOM treatments. SOIL–POLL was the only treatment found to present significant correlation between BA and BP (*r* = 0.66; *p* = 0.002).

Bacterial production and DOC were negatively correlated in the control (*r* = -0.70, *p* = 0.001), while these two factors were positively correlated in SOIL-EX treatment (*r* = 0.66, *p* = 0.003). On the other hand, comparable correlations were considerably weaker and not significant in treatments SOIL–POLL and POLL (*r*-values 0.23 and -0.15, respectively; *p*-values 0.37 and 0.54, respectively). BP showed significant positive correlations with Ptot in all treatments except for SOIL–POLL (*r* = 0.31, *p* = 0.21), though non-tDOM treatments showed stronger correlations (*r* > 0.73, *p* < 0.001). However, while in non-tDOM treatments this correlation is explained by a simultaneous, progressive decrease of Ptot and BP, in tDOM treatments the correlations seem to lie in overall increasing values of these two variables (Figure 2B and Supplementary Figure S3C).

Primary production substantially declined throughout the experiment in all treatments (Supplementary Figure S3A), although SOIL-EX treatment showed higher overall values compared to the non-tDOM treatments (*p* = 0.008, $\eta_{p}^{2}$ = 0.21). On the other hand, Chl-*a* differed significantly among treatments (*p* < 0.0001, $\eta_{p}^{2}$ = 0.67), being markably higher in tDOM treatments, especially in SOIL-EX (Supplementary Figure S3B). Both PP and Chl-*a* showed strong positive correlations with BP in non-tDOM treatments (*r* = 0.65–0.85), with Chl-*a* also showing a significant correlation in SOIL-EX treatment (*r* = 0.66, *p* = 0.003).

### Bacterial Community Structure

**FIGURE 3:**
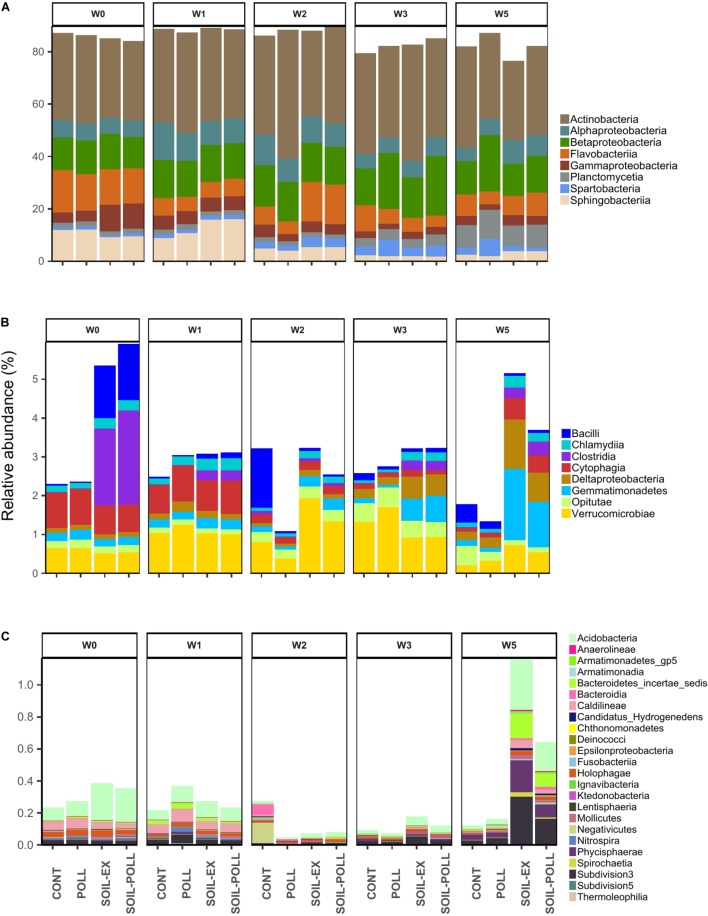
Relative abundance of bacterial taxa (at Class level) from the different treatments and different sampling times. According to their relative abundances, the bacterial taxa were classified into **(A)** abundant taxa (>1%). **(B)** Moderate taxa (between 0.1 and 1%), and **(C)** rare taxa (<0.1%). Unclassified OTUs were discarded.

A total of 11,631,371 high-quality sequences remained after quality control, trimming and denoizing, with an average of 176,233 ± 36,625 sequences per sample, which resulted in a total of 11,321 OTUs recorded after clustering at 97% similarity level. In order to discriminate and visualize the structure of the bacterial communities, the taxa were divided based on their relative contribution to the overall community using the following thresholds set at class level: >1% as abundant taxa, between 0.1% and 1% as moderate taxa, and <0.1% as rare taxa (Figure 3 and Table 1). Similar classifications have been used previously (e.g., Lindh et al., 2015; Yan et al., 2017). The most abundant classes were *Actinobacteria*, *Betaproteobacteria*, *Flavobacteriia*, *Alphaproteobacteria*, *Sphingobacteriia*, and *Gammaproteobacteria*, comprising 67–86% of the overall relative abundance (Figure 3A). Although some fluctuation was observed, their relative abundances in general remained relatively stable throughout the experimental time and across treatments. Nonetheless, *Gammaproteobacteria* and *Sphingobacteriia* generally showed higher relative abundances during the first weeks, particularly in the tDOM-amended treatments. Moderately abundant taxa were composed of eight major classes (Figure 3B). Differences between tDOM and non-tDOM treatments were apparent in this category, for example, *Bacilli* and *Clostridia* at the beginning of the experiment, or *Deltaproteobacteria* and *Gemmatimonadetes* at weeks 3 and 5. Rare taxa encompassed a much larger selection of bacterial classes than the more abundant taxa (Figure 3C), and differences between tDOM and non-tDOM treatments were observed, especially at week 5. *Acidobacteria*, *Bacteroidetes incertae sedis*, *Phycisphaerae*, and *Subdivision_3* represent notable examples.

**Table 1:** Adjusted *p*-values from the GLM on bacterial classes found in the bacterial communities from the different treatments.

		CONT/SOIL-EX		CONT/SOIL-POLL		CONT/POLL		SOIL-EX/SOIL-POLL		SOIL-EX/POLL		SOIL-POLL/POLL	
							
		Treatment	Sampling	Treatment	Sampling	Treatment	Sampling	Treatment	Sampling	Treatment	Sampling	Treatment	Sampling
	Overall effects	***0.004***	***0.010***	***0.018***	***0.007***	0.279	***0.014***	0.646	***0.006***	***0.004***	***0.004***	***0.003***	***0.002***
Abundant taxa (>1%)	*Actinobacteria*	0.962	0.801	0.983	0.803	1	0.408	1	0.197	0.807	0.443	0.905	0.246
	*Alphaproteobacteria*	1	***0.029***	1	0.168	1	***0.021***	1	***0.027***	0.941	***0.005***	1	0.063
	*Betaproteobacteria*	1	1.000	0.958	0.351	0.257	0.408	0.957	***0.031***	0.317	0.291	0.998	***0.004***
	*Flavobacteriia*	1	0.375	1	0.343		***0.021***	1	***0.025***		***0.020***		***0.027***
	*Gammaproteobacteria*	0.864	0.366	0.989	0.242	0.517	***0.022***	1	***0.026***	0.542	***0.004***	0.713	***0.004***
						0.921				0.317		0.435	
	*Planktomycetia*	1	***0.027***	1	***0.011***	1	***0.021***	1	***0.025***	0.965	***0.004***	1.000	***0.004***
	*Spartobacteria*	1	***0.028***	1	***0.013***	0.649	***0.021***	1	***0.021***	0.650	***0.009***	0.747	***0.010***
	*Sphingobacteriia*	0.999	***0.027***	1	***0.012***	1	***0.021***	1	***0.025***	0.943	***0.004***	1	***0.004***
Moderate taxa (0.1 = 1%)	*Bacilli*	0.907	0.828	1	0.768	0.180	0.070	1	***0.025***	0.317	0.084	0.210	0.104
	*Clostridia*	***0.013***	0.828	***0.004***	0.686	0.374	0.887	1	***0.025***	***0.003***	***0.004***	***0.004***	***0.004***
	*Chlamydiia*	0.147	0.458	0.052	0.310	1	***0.022***	1	0.057	***0.004***	***0.006***	***0.009***	***0.004***
	*Cytophagia*		***0.027***	1	***0.012***	1	***0.021***	1	***0.025***	0.972	***0.004***	1	***0.004***
	*Deltaproteobacteria*	0.999	***0.029***	0.324	***0.017***	1	0.070	1	***0.025***		***0.006***	0.390	***0.015***
		0.335								0.317			
	*Gemmatimonadetes*	***0.016***	0.652	***0.054***	0.834	0.977	0.369	1	***0.025***	***0.004***	0.206	***0.004***	0.681
	*Opitutae*	0.962	0.340	0.933	0.335	1	***0.027***	1	***0.025***	0.941	***0.004***	0.943	***0.004***
	*Verrucomicrobiae*	0.975	0.495	1	0.280	1	***0.021***	0.993	***0.026***	0.952	0.206	1	0.083
Rare taxa (<0.1%)	*Acidobacteria_Gp1*	***0.013***	0.901	***0.005***	0.803	1	0.863	1	***0.025***	***0.003***	***0.032***	***0.004***	***0.040***
	*Acidobacteria_Gp2*	***0.014***	0.604	***0.004***	0.698	0.977	0.887	1	***0.027***	***0.014***	0.305	***0.017***	0.317
	*Ktedonobacteria*	***0.013***	0.828	***0.004***	0.650	1	0.887	1	0.146	***0.003***	0.485	***0.004***	0.253
	*Negativicutes*	0.864	0.467	0.976	0.507	0.829	0.408	0.968	0.254	0.288	0.545	0.210	0.888
	*Phycisphaerae*	0.625	***0.028***	0.977	***0.012***	1	***0.021***	0.957	***0.025***	0.542	***0.004***	1	***0.005***
	*Candidatus_Hydrogenedens*	0.806	0.467	0.939	0.698	1	0.175	1	***0.026***	0.398	0.108	0.665	0.194
	*Subdivision 3*	0.261	***0.029***	0.498	***0.013***	1	***0.025***	0.979	***0.025***	0.324	***0.005***	0.863	***0.004***


*The GLM was applied in pairwise comparisons between the treatments. Bacterial classes are classified according to their relative abundance in the communities into abundant, moderate, and rare taxa. The factor “Sampling” refers to changes throughout the experimental time. Overall effects from the different pairwise comparisons are shown. Significant *p*-values are shown in bold. The corresponding LR values are shown in Supplementary Table S3.*

### Diversity of Bacterial Communities

**FIGURE 4:**
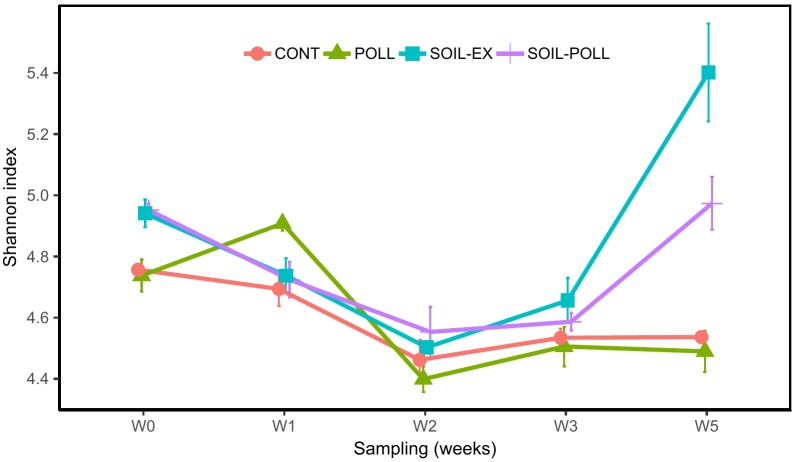
Development of biodiversity in bacterial communities (Shannon’s diversity index) from the different treatments (CONT, control; POLL, pollutants addition; SOIL-EX, tDOM addition; SOIL–POLL, tDOM + pollutants addition) throughout the experiment. Error bars represent the standard deviation (*n* = 3).

The diversity (Shannon’s diversity index) of the communities decreased from the outset of the experiment until week 2 in all treatments, after which a divergence between tDOM and non-tDOM treatments (increasing or plateauing, respectively) was recorded (Figure 4). Significant differences were found between different treatments across the overall experimental period, with tDOM treatments showing higher diversity than non-tDOM treatments (Figure 4 and Supplementary Table S2). On the other hand, no overall significant differences were found between pollutant and non-pollutant containing treatments. However, analysis of OTU composition from week 5 alone (representative of the outcome of persistent exposure to the pollutant mixture) indicated significant differences between treatments CONT and POLL, and between SOIL-EX and SOIL–POLL (Table 3).

### Bacterial Classes Responsible for Structural Change Over the Duration of the Experiment

**FIGURE 5:**
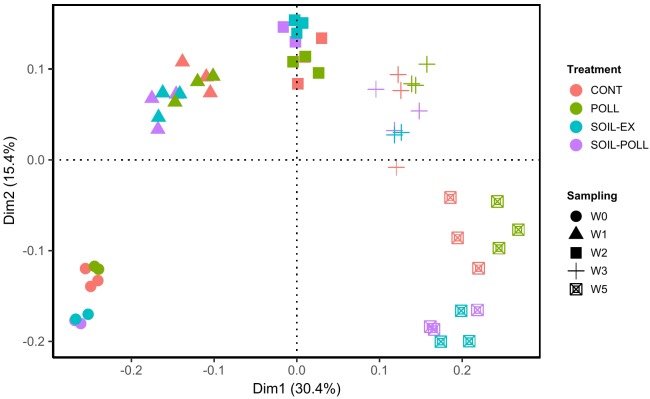
Principal coordinate analysis (PCoA) of bacterial communities from the different treatments and from different sampling times (weeks). Similarities were based on the Bray–Curtis distance. The individual samples are coded according to a color (treatment) and a shape (sampling time).

In order to statistically study the changes in community composition between treatments and throughout the experimental period, a GLM was applied in pairwise comparisons at class level. The GLM analyses showed strong and significant differences (*p*-values < 0.01) between the community compositions based on sampling occasion (i.e., a temporal effect) and due to the addition of tDOM for all pair-wise comparisons (Table 1 and Supplementary Table S3, overall effects). However, this overall analysis gave no indication of a pollutant-based treatment effect (Table 1, overall effects). Similarly, a PCoA indicated a tendency for closer clustering of samples from non-tDOM treatments on the one hand, and tDOM treatments on the other (Figure 5), although the temporal progression (i.e., sampling time) was clearly a more dominant factor.

The GLM highlighted bacterial taxa that significantly contributed to the differences observed between communities over time and between treatments. Within the abundant classes, *Actinobacteria* did not significantly contribute to temporal or treatment differences. Other bacterial classes among the abundant group, such as *Alphaproteobacteria*, *Betaproteobacteria*, *Planctomycetia*, or *Spartobacteria*, did not show significant differences between treatments. However, they did show significant differences according to sampling occasion in most of the pairwise comparisons (Table 1). This is in line with the relative abundances shown in Figure 3A, where *Alphaproteobacteria* increased in mid-experiment, *Planctomycetia* generally increased toward the end of the experiment, and *Spartobacteria* showed higher relative abundances from week 2 onward. *Flavobacteria*, *Gammaproteobacteria*, and *Sphingobacteria* classes showed no significant differences by treatments overall, although significant temporal differences were found (Table 1). However, similar relative abundances of some classes on certain sampling weeks showed pairing between the two tDOM treatments and pairing of the non-tDOM treatments, for example *Flavobacteria* at week 2 (Figure 3A).

Some of the moderate and rare classes, such as *Clostridia* and *Acidobacteria*, showed significant differences between tDOM and non-tDOM treatments. Other classes, such as *Cytophagia*, *Deltaproteobacteria*, *Phycisphaerae*, and *Subdivision_3*, presented significant temporal differences (Figures 3B,C and Table 1).

### Specific OTUs Contributing to Significant Community Structure Changes, and Correlations With Physicochemical Aspects and Pollutants

**FIGURE 6:**
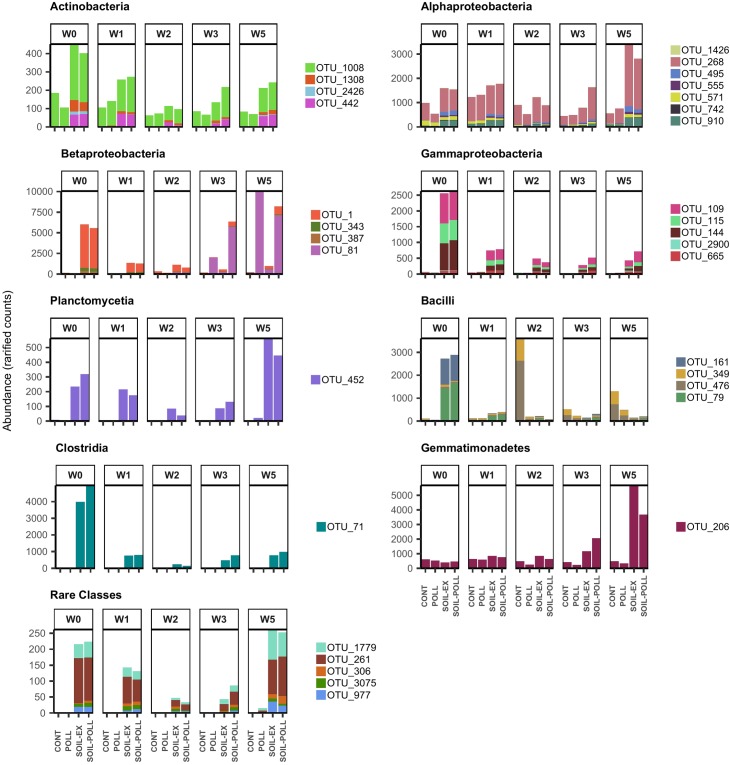
Abundance (rarified counts) of OTUs from GLM model contributing to the significant differences observed between communities from different treatments and sampling times. The OTUs are classified according to their bacterial taxonomy at class level. Specific taxonomic classification corresponding to these OTUs is shown in Table 2.

Individual OTUs contributing to the significant differences observed between communities from different treatments were highlighted using a GLM approach at the OTU level. Among the 544 OTUs recorded, only 32 showed overall significant differences between treatments (Figure 6 and Table 2). Such OTUs were generally in low abundance, although they mostly belonged to abundant classes such as *Actinobacteria*, *Alphaproteobacteria*, *Betaproteobacteria*, *Gammaproteobacteria*, and *Planctomycetia*. Six of these OTUs belonged to moderate classes (*Bacilli*, *Clostridia*, and *Gemmatimonadetes*) and five to rare classes (*Acidobacteria_Gp1* and _*Gp2*, *Ktedonobacteria*, and *Negativicutes*). Overall, the differences generally lay in higher abundances of these OTUs occurring in tDOM treatments, which is in line with higher Shannon’s index values from these treatments. However, one exception was OTU_81 (*Betaproteobacteria*), that appeared to respond to the presence of pollutants. This OTU remained in low abundance (52–223 reads; rarefied counts) until week 2 in all treatments, after which it increased to as many as 10,015 reads in polluted treatments toward the end of the experiment. It is worth noting that the five OTUs belonging to rare classes were found to be present only in tDOM treatments, which suggests that their presence was specifically attributed to the addition of soil extract (Figure 6). In addition, these OTUs were indeed found in samples from the soil extract (Supplementary Figure S4).

**Table 2:** OTUs that significantly contributed to the community composition differences observed between treatments (GLM analysis).

			Adjusted *p*-value					
			
OTU_ID	Class	Taxon	CONT	CONT	CONT	SOIL-EX	SOIL-EX	SOIL–POLL
					
			SOIL-EX	SOIL–POLL	POLL	SOIL–POLL	POLL	POLL
OTU_1	*Betaproteobacteria*	*Janthinobacterium*	**0.008**	**0.014**	>0.05	>0.05	**0.001**	**0.001**
OTU_71	*Clostridia*	*Clostridium_sensu_stricto*	**0.001**	**0.007**	>0.05	>0.05	**0.001**	**0.001**
OTU_79	*Bacilli*	*Paenibacillus*	**0.002**	**0.008**	>0.05	>0.05	**0.001**	**0.001**
OTU_81	*Betaproteobacteria*	*Methylophilus*	>0.05	**0.014**	**0.038**	>0.05	>0.05	>0.05
OTU_109	*Gammaproteobacteria*	*Buttiauxella*	**0.001**	**0.006**	>0.05	>0.05	**0.001**	**0.001**
OTU_115	*Gammaproteobacteria*	*Enterobacteriaceae*	**0.002**	**0.007**	>0.05	>0.05	**0.002**	**0.001**
OTU_144	*Gammaproteobacteria*	*Yersinia*	**0.002**	**0.008**	>0.05	>0.05	**0.001**	**0.001**
OTU_161	*Bacilli*	*Bacillales*	**0.032**	**0.025**	>0.05	>0.05	**0.003**	**0.001**
OTU_206	*Gemmatimonadetes*	*Gemmatimonas*	**0.048**	**0.045**	>0.05	>0.05	**0.010**	**0.003**
OTU_261	*Acidobacteria_Gpl*	*Gp1*	**0.001**	**0.006**	>0.05	>0.05	**0.002**	**0.001**
OTU_268	*Aiphaproteobacteria*	*Rhizobiales*	>0.05	**0.017**	>0.05	>0.05	**0.012**	**0.001**
OTU_306	*Negativicutes*	*Veillonellaceae*	>0.05	>0.05	>0.05	>0.05	**0.020**	**0.017**
OTU_343	*Betaproteobacteria*	*Silvimonas*	**0.001**	**0.007**	>0.05	>0.05	**0.002**	**0.001**
OTU_349	*Bacilli*	*Lactococcus*	>0.05	**0.045**	>0.05	>0.05	>0.05	>0.05
OTU_387	*Betaproteobacteria*	*Burkholderia*	**0.010**	**0.014**	>0.05	>0.05	**0.001**	**0.001**
OTU_442	*Actinobacteria*	*Thermomonosporaceae*	**0.001**	**0.007**	>0.05	>0.05	**0.001**	**0.001**
OTU_452	*Planctomycetia*	*Aquisphaera*	**0.001**	**0.001**	>0.05	>0.05	**0.001**	**0.001**
OTU_476	*Bacilli*	*Streptococcus*	**0.047**	**0.045**	>0.05	>0.05	>0.05	>0.05
OTU_495	*Aiphaproteobacteria*	*Bradyrhizobium*	**0.011**	**0.014**	>0.05	>0.05	**0.002**	**0.001**
OTU_555	*Aiphaproteobacteria*	*Acidocella*	**0.001**	**0.006**	>0.05	>0.05	**0.002**	**0.001**
OTU_665	*Gammaproteobacteria*	*Aquicella*	**0.034**	**0.025**	>0.05	>0.05	**0.003**	**0.001**
OTU_742	*Aiphaproteobacteria*	*Rhodomicrobium*	**0.002**	**0.011**	>0.05	>0.05	**0.002**	**0.004**
OTU_910	*Aiphaproteobacteria*	*Acetobacteraceae*	>0.05	>0.05	>0.05	>0.05	**0.027**	**0.017**
OTU_571	*Aiphaproteobacteria*	*Beijerinckiaceae_unclassified*	>0.05	>0.05	>0.05	>0.05	**0.049**	**0.047**
OTU_977	*Acidobacteria_Gpl*	*Granulicella*	**0.006**	**0.007**	>0.05	>0.05	**0.003**	**0.001**
OTU_1008	*Actinobacteria*	*Conexibacter*	>0.05	**0.048**	>0.05	>0.05	>0.05	>0.05
OTU_1308	*Actinobacteria*	*Aciditerrimonas*	**0.005**	**0.011**	>0.05	>0.05	**0.050**	**0.043**
OTU_1426	*Aiphaproteobacteria*	*Beijerinckia*	**0.048**	**0.011**	>0.05	>0.05	**0.002**	**0.001**
OTU_1779	*Acidobacteria_Gp2*	*Gp2*	**0.002**	**0.009**	>0.05	>0.05	**0.050**	**0.043**
OTU_2426	*Actinobacteria*	*lamia*	>0.05	>0.05	>0.05	>0.05	>0.05	>0.05
OTU_2900	*Gammaproteobacteria*	*Pseudomonadaceae*	>0.05	>0.05	>0.05	>0.05	**0.048**	**0.046**
OTU_3075	*Ktedonobacteria*	*Ktedonobacter*	**0.010**	**0.008**	>0.05	>0.05	**0.005**	**0.001**


*Adjusted *p*-values are shown for every pairwise comparison. The taxonomic information provided is based on taxa names at class level and at the lowest level of classification (Silva v. 123) reached in every case. Significant *p*-values are shown in bold. The corresponding LR values are shown in Supplementary Table S4.*

Pearson’s correlations between specific OTUs (those identified by the GLM analysis) and an array of physicochemical and biological variables measured during the mesocosm experiment indicated strong and significant correlations between the abundance of certain OTUs and DOC concentration, PP, and Chl-*a*; especially in the tDOM treatment mesocosms (Supplementary Table S5). While the correlations with PP were mainly positive, the correlations with DOC were generally negative. However, in many cases the strong negative correlations with DOC relied on higher numbers of these OTUs during week 0 (Figure 6 and Supplementary Figure S5), after which their abundance stabilized or decreased, while DOC concentration continued rising due to further (smaller) soil additions. Among the bacterial taxa responding positively to elevated tDOM, some OTUs showed significant correlations with both DOC and nutrients (Ptot and Ntot), such as OTU_206, OTU_268, OTU_452, and OTU_495 (Supplementary Table S5). On the contrary, most of these OTUs showed significant correlations with DOC but not with nutrients (e.g., OTU_1, OTU_71, OTU_79, OTU_109, etc.).

Correlations between OTU abundance and concentration of the different pollutants added to the mesocosms highlighted a number of relevant interactions between some OTUs and pollutants (Supplementary Figure S6 and Supplementary Table S6). Some OTUs, such as OTU_79, OTU_144, or OTU_387, showed strong and significant correlations with several pollutants almost exclusively in the SOIL–POLL treatment, though it must be noted that these OTUs also showed strong correlations with DOC. On the other hand, OTUs, such as OTU_206, OTU_268, OTU_571, and OTU_1008, showed significant correlations with different pollutants, almost exclusively under POLL treatment. Certain OTUs (e.g., OTU_81, OTU_115, and OTU_3075) showed significant correlations under both pollutant addition treatments. Finally, some OTUs did not show any significant correlation with the concentrations of any pollutants in the added pollutant cocktail (e.g., OTU_442 and OTU_495). Most of the significant correlations between specific OTUs and pollutant concentrations were positive, though certain OTUs such as OTU_81, OTU_261, and OTU_343 showed negative correlations (Supplementary Figure S6 and Supplementary Table S6). However, it must be noted that OTU_81 showed increased abundance at the latter stages of the experiment in both pollutant addition treatments (Figure 6).

### Community Structure Differences Driven by Extended Exposure to Organic Pollutants

**FIGURE 7:**
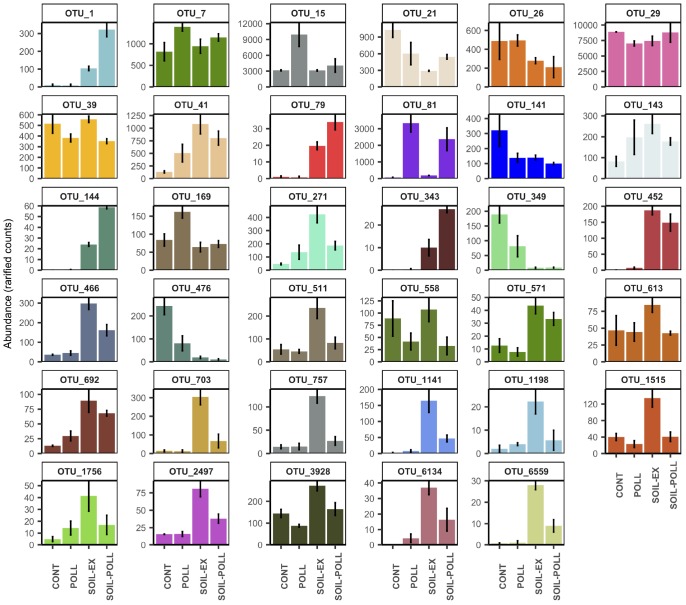
Abundance (rarified counts) of OTUs found at week 5 showing significant differences in abundance (one-way ANOVA) between polluted and non-polluted treatments (i.e., CONT vs. POLL and SOIL-EX vs. SOIL–POLL). Error bars represent the standard deviation (*n* = 3). Specific taxonomic classification corresponding to these OTUs is shown in Table 3.

At the end of the experiment (i.e., at week 5), a number of OTUs showing significant differences in abundance between polluted and non-polluted treatments (i.e., CONT vs. POLL and SOIL-EX vs. SOIL–POLL) were identified (Figure 7 and Table 3). OTUs containing less than 100 reads in total (from all replicates) were discarded, leaving 13 OTUs that showed significant differences between treatments CONT and POLL, and 25 OTUs between treatments SOIL-EX and SOIL–POLL (Figure 7 and Table 2). Eight of thirteen OTUs showed higher abundance in the POLL treatment, whereas only 6 of 25 did so in the SOIL–POLL treatment. Of these, only OTU_81 showed significant differences in both pair-wise comparisons (Figure 7).

**Table 3:** OTUs found at week 5 showing significant differences in abundance between the different treatments (Tukey’s Honest Significant Differences on rarefied counts).

			Adjusted *p*-value					
			
OTU_ID	Class	Taxon	CONT	SOIL-EX	CONT	SOIL–POLL	SOIL-EX	CONT
					
			POLL	SOIL–POLL	SOIL-EX	POLL	POLL	SOIL–POLL
OTU_1	*Betaproteobacteria*	*Janthinobacterium*	0.823	**0.006**	**0.002**	**0.002**	**0.002**	**0.002**
OTU_7	*Alphaproteobacteria*	*Gemmobacter*	**0.043**	0.328	0.663	0.134	0.078	0.220
OTU_15	*Betaproteobacteria*	*Methylophilaceae*	**0.040**	0.504	0.899	0.086	**0.040**	0.513
OTU_21	*Gammaproteobacteria*	*Pseudomonas*	**0.048**	**0.004**	**0.006**	0.790	0.196	**0.027**
OTU_26	*Sphingobacteriia*	*Saprospiraceae*	0.979	0.583	**0.047**	0.087	**0.032**	0.285
OTU_29	*Actinobacteria*	*llumatobacter*	**0.009**	0.477	0.127	0.334	0.651	0.964
OTU_39	*Betaproteobacteria*	*Alcaligenaceae*	0.239	**0.005**	0.682	0.522	**0.021**	0.149
OTU_41	*Alphaproteobacteria*	*Rhodobacteraceae*	**0.042**	0.308	**0.009**	0.253	0.093	**0.009**
OTU_79	*Bacilli*	*Paenibacillus*	0.725	**0.053**	**0.001**	**0.002**	**0.001**	**0.002**
OTU_81	*Betaproteobacteria*	*Methylophilus*	**0.004**	**0.033**	**0.003**	0.328	**0.004**	**0.028**
OTU_141	*Betaproteobacteria*	*Oxalobacteraceae*	0.177	**0.043**	**0.041**	0.293	0.963	0.111
OTU_143	*Verrucomicrobiae*	*Verrucomicrobiaceae*	**0.043**	0.164	**0.026**	0.824	0.533	**0.032**
OTU_144	*Gammaproteobacteria*	*Yersinia*	0.374	**<0.001**	**0.000**	**<0.001**	**<0.001**	**<0.001**
OTU_169	*Alphaproteobacteria*	*Loktanella*	**0.031**	0.630	0.407	**0.010**	**0.010**	0.587
OTU_271	*Planctomycetia*	*Planctomyces*	0.176	**0.030**	**0.004**	0.466	**0.027**	**0.013**
OTU_343	*Betaproteobacteria*	*Silvimonas*	0.374	**0.012**	**0.043**	**<0.001**	**0.045**	**<0.001**
OTU_349	*Bacilli*	*Lactococcus*	**0.032**	0.806	**0.003**	0.112	0.110	**0.004**
OTU_452	*Planctomycetia*	*Aquisphaera*	0.053	**0.042**	**<0.001**	**0.006**	**<0.001**	**0.005**
OTU_466	*Subdivisions*	*Subdivisions*	0.484	**0.032**	**0.001**	**0.019**	**0.002**	**0.012**
OTU_476	*Bacilli*	*Streptococcus*	**0.031**	0.171	**0.004**	0.098	**0.039**	**0.003**
OTU_511	*Deltaproteobacteria*	*Peredibacter*	0.706	**0.043**	**0.023**	0.234	**0.015**	0.442
OTU_558	*Alphaproteobacteria*	*Novosphingobium*	0.303	**0.032**	0.697	0.736	0.096	0.235
OTU_571	*Alphaproteobacteria*	*Beijerinckiaceae*	0.460	**0.048**	**0.020**	**0.012**	**0.007**	**0.046**
OTU_613	*Actinobacteria*	*Pseudoclavibacter*	0.932	**0.021**	**0.046**	0.910	**0.034**	0.866
OTU_692	*Alphaproteobacteria*	*Hoeflea*	**0.045**	0.353	**0.018**	**0.016**	**0.031**	**<0.001**
OTU_703	*Deltaproteobacteria*	*Bdellovibrio*	0.786	**0.013**	**0.002**	0.210	**0.002**	0.222
OTU_757	*Gammaproteobacteria*	*Glaciecola*	0.937	**0.006**	**0.002**	0.356	**0.003**	0.282
OTU_1141	*Gammaproteobacteria*	*Hahella*	0.199	**0.037**	**0.011**	**0.025**	**0.013**	**0.013**
OTU_1198	*Gammaproteobacteria*	*Arenimonas*	0.288	**0.024**	**0.021**	0.718	**0.027**	0.463
OTU_1515	*Candidatus_Saccharibacteria*	*Candidatus_Saccharibacteria*	0.227	**0.019**	**0.016**	0.280	**0.009**	0.965
OTU_1756	*Alphaproteobacteria*	*Altererythrobacter*	**0.047**	**0.046**	**0.049**	0.801	**0.039**	0.221
OTU_2497	*Deltaproteobacteria*	*Bacteriovoracaceae*	0.929	**0.032**	**0.005**	**0.041**	**0.006**	**0.027**
OTU_3928	*Parcubacteria*	*Parcubacteria*	**0.046**	**0.046**	**0.013**	0.064	**0.002**	0.595
OTU_6134	*Caldilineae*	*Litorilinea*	0.203	**0.032**	**0.001**	0.201	**0.004**	**0.025**
OTU_6559	*Deltaproteobacteria*	*Nannocystaceae*	0.768	**0.006**	**<0.001**	0.059	**<0.001**	**0.046**


*Adjusted *p*-values from all possible pair-wise comparisons are shown. For each OTU, class, and taxon names are provided, the latter referring to the lowest taxonomic level successfully reached by SILVA database (i.e., genus, family, order, or class). Significant p-values are shown in bold.*

In order to find out whether these OTUs were driven by soil extract addition and not only by the presence of pollutants, the same OTUs were studied by analysis of variance between tDOM and non-tDOM treatments at week 5 (i.e., CONT vs. SOIL-EX and POLL vs. SOIL–POLL) (Figure 7 and Table 2). OTU_7, OTU_15, and OTU_169 showed the highest abundance in treatment POLL compared with the other treatments. OTU_81 showed significantly higher abundance in both polluted treatments compared with the non-polluted treatments. On the other hand, OTU_29 showed lower abundance in treatment POLL compared with the control. OTU_41 and OTU_692 showed significantly higher abundance in treatments with increased DOC concentration (i.e., treatments POLL, SOIL-EX, and SOIL–POLL), while others, such as OTU_21, OTU_349, and OTU_476, showed lower abundance in these treatments compared with the control. Many of the OTUs from this analysis (17 out of 35) presented the highest abundances in treatment SOIL-EX compared with the rest of treatments (e.g., OTU_271, OTU_613, OTU_703, OTU_757, etc.), showing significantly lower abundances in treatment SOIL–POLL compared with treatment SOIL-EX. On the other hand, OTU_1, OTU_79, OTU_144, and OTU_343 showed the highest abundances in treatment SOIL–POLL compared to the rest, including treatment SOIL-EX.

## Discussion

### Impact of tDOM on Bacterial Community Structure

Allochthonous organic matter inputs have been shown to promote bacterial metabolism and lead to changes in bacterial community structure (Crump et al., 2003; Figueroa et al., 2016; Traving et al., 2017). In the present study, increased tDOM concentration (in the form of soil extract) had a measurable effect on diversity and community structure, altering the bacterioplankton community present in the starting mesocosm waters (natural Baltic Sea waters). The addition of soil extract led to higher BP, higher total BA, and higher bacterial α-diversity (Shannon’s diversity). However, the relative abundances of the major bacterial classes identified in this study were not significantly affected by the addition of tDOM, remaining relatively stable between the different treatments (Figure 3A). Classes such as *Actinobacteria*, *Alphaproteobacteria*, *Betaproteobacteria*, *Spartobacteria*, or *Flavobacteriia* have been widely described to be common and abundant members of bacterial communities in marine waters, particularly in the Baltic Sea (Lindh et al., 2015, 2016), which is consistent with the high degree of resilience and redundancy of these bacterial groups under environmental disturbance (Allison and Martiny, 2008; Shade et al., 2012). The greatest differences in community structure at class level between the different treatments were mainly observed in moderately abundant and rare bacterial classes. For example, relative abundance of *Acidobacteria*, *Cytophagia*, and *Deltaproteobacteria* generally correlated positively with increased tDOM concentrations (Figures 3B,C), and as noted in other studies (Kersters et al., 2001; Pedrós-Alió, 2012) shifts in the non-abundant bacterial taxa were observed.

Despite the relative stability of the abundant and many moderately abundant classes, a number of OTUs belonging to these classes were positively affected by tDOM addition. Bacteria belonging to the orders *Rhizobiales* (e.g., *Bradyrhizobium* spp., OTU_495; and OTU_268), *Enterobacteriales* (e.g., *Buttiauxella* spp., OTU_109 and *Yersinia* spp., OTU_144), *Solirubrobacterales* (*Conexibacter* ssp., OTU_1008), *Burkholderiales* (*Janthinobacterium* spp., OTU_1), *Bacillales* (*Paenibacillus* spp., OTU_79 and OTU_161), and *Clostridiales* (*Clostridium sensu stricto* ssp., OTU_71) were boosted by the addition of soil extract (Figure 6), before stabilizing at lower abundances during the later stages of the experiment. These bacterial classes likely represent members of the soil bacterial community that entered the system due to soil extract addition, though determining survival or any particular function within the system would require further specific studies. On the other hand, bacteria belonging to the orders *Gemmatimonadaceae* (*Gemmatimonas* spp., OTU_206), *Rhodobacterales* (OTU_41), *Actinomycetales* (*Pseudoclavibacter* spp., OTU_613), *Planctomycetales* (*Planctomyces* spp., OTU_271), or *Alteromonadales* (*Glacieola* spp., OTU_757) showed a positive response to tDOM addition, increasing in relative abundance at later stages of the experiment (Figures 6, 7). These differing trends suggest different dynamics in bacterial activity from different members in the same community, with some bacterial taxa displaying higher response capabilities than others under elevated tDOM, which has been observed in similar studies (Traving et al., 2017). Most of these bacterial taxa have previously been shown to respond positively to tDOM. For example, the *Alphaproteobacteria* orders *Rhodobacterales* and *Rhizobiales* have been described to metabolize high and low molecular weight organic matter and various aromatic compounds, and to inhabit both terrestrial and aquatic environments (Kersters et al., 2001; Li et al., 2012). The *Gammaproteobacteria* genus *Glacieola* has been described to be a non-abundant member of bacterioplankton communities capable of growing under increased DOM levels (Sipler et al., 2017; von Scheibner et al., 2017). The genus *Gemmatimonas* (*Gemmatimonadetes* class) responded positively to long-term exposure to increased concentration of DOC (i.e., after 5 weeks), which has been also described in a similar study (Traving et al., 2017). On the other hand, although most of the bacteria significantly affected by the addition of tDOM showed a positive response, we also found some bacteria that responded negatively to increased tDOM, such as *Pseudomonas* ssp. (OTU_21), *Lactococcus* ssp. (OTU_349), *Streptococcus* ssp. (OTU_476), and an unclassified member of the family *Oxalobacteraceae* (OTU_141). These bacteria showed the highest abundances in the control mesocosms and remarkably decreased in abundance in the mesocosms with tDOM addition.

Dissolved organic matter characteristics and inputs can influence bacterial activity (Berggren and del Giorgio, 2015; Rowe et al., 2018), even if production is only temporarily elevated (Figueroa et al., 2016). Furthermore, allochthonous DOM can influence the bacterial community (Judd et al., 2006; Logue et al., 2016), and the processing of the DOM can have consequences for ecosystem function and global biogeochemical cycles (Jiao et al., 2010; Benner and Amon, 2015). In this study, patterns of BP in mesocosms receiving tDOM addition differed from their respective controls, with a secondary peak developing in the latter stages of the experiment. This corresponded with a notable increase of Ptot and DIP after 2 weeks in the mesocosms that also took place only in mesocosms with tDOM addition. The release of inorganic phosphorus as soluble reactive phosphorus (SRP) from anoxic sediments to the water column has been described in both lakes and brackish waters such as the Baltic Sea (Stigebrandt et al., 2014; Wu et al., 2015). In the present experiment, we observed a decrease in phytoplankton abundance during the first 2 weeks (unpublished data) and an enhanced sedimentation in the mesocosms with tDOM addition resulting in sinking particles and aggregates from the water column (Ripszam et al., 2015b). The sedimentation of such particles created a visible bottom layer within the mesocosms, consisting of organic matter from phytoplankton detritus, zooplankton fecal pellets, and particles from the added tDOM itself. It appears likely that the anoxic and micro-oxic environments that would have formed contributed greatly to the *P* cycling in the experimental systems. Under anoxic conditions, the phosphorous stored in the sedimented particles can be released to the water column as DIP or SRP due to microbial processes carried out by some bacteria, such as sulfate-reducing bacteria, leading to an internal loading of phosphorus and thus an elevation of both the DIP and the total phosphorus in the water column (Palmer-Felgate et al., 2011; Sinkko et al., 2011). The internal phosphorus loading is a phenomenon increasingly studied in the Baltic Sea (Stigebrandt et al., 2014). Furthermore, such processes have been identified to take place even within aggregates and particles formed within the water column itself (Ploug et al., 1997; Riemann and Azam, 2002). In the present study, microbial communities from sedimented material itself were not analyzed. However, we found some sulfate-reducing bacteria present in low abundance especially in the soil extract utilized, such as *Desulfovibrio*, *Desulfosporosinus*, *Desulfopila*, *Desulfofaba*, and *Desulfobulbus* (data not shown), which have been previously described in the Baltic Sea (Korneeva et al., 2015). Within the oxygen depleted microzones generated in the bottom of the mesocosms receiving tDOM addition, the metabolism of these specific bacteria could have been enhanced, enabling them to generate the increased DIP and Ptot concentrations observed. Since *P* is generally the limiting nutrient in the region where study waters were collected (Tamminen and Andersen, 2007; Andersson et al., 2015), this process would clarify why bacterial communities, now flushed with newly released DIP, were able to have a second peak of production in the later stages of the experiment only in those mesocosms receiving tDOM addition.

### Effects of Pollutants and Co-effects of tDOM Addition

The effects of organic pollutants on bacterial communities were generally less pronounced than the effects observed due to tDOM addition. However, differences at the OTU level between polluted and non-polluted treatments were recorded. Pollutant addition appeared to have little impact on BP and Shannon’s diversity in the CONT–POLL comparison, but mesocosms with tDOM addition were apparently negatively impacted by pollutant addition in the latter stages of the experiment (Figures 2, 4). In general, the differences in bacterial diversity creating this latter pattern mainly resulted from higher abundances of some OTUs in SOIL-EX compared to SOIL–POLL, such as OTU_39 (*Alcaligenaceae* family), OTU_271 (*Planctomyces* spp.), or OTU_613 (*Pseudoclavibacter* spp.), among others (Figure 7). This could indicate some form of direct toxicity due to the pollutant addition and would explain why the strong recovery of BP in the SOIL-EX treatment was inhibited in the SOIL–POLL treatment despite the same additions of tDOM. The lower bacterial diversity observed in the SOIL–POLL treatment, as opposed to the SOIL-EX treatment, was generally due to the overall decrease in abundance of rare bacteria (>1% relative abundance). Similarly, other studies have pointed out the importance of fluctuations of rare bacteria on the diversity of bacterial communities in the presence of organic pollutants (Sun et al., 2012).

On the other hand, we detected some combined effects of tDOM and pollutants. Four major trends (Figure 7) were detected as a result of the co-effects of pollutant and tDOM addition. Thus, pollutant addition had a positive effect on certain bacteria, which was removed by the addition of tDOM, possibly due to the attachment of the organic pollutants to DOC and other particles (Ripszam et al., 2015a), preventing these bacteria from utilizing them. This trend was observed in, for example, two members of the family *Rhodobacteraceae* (*Gemmobacter* ssp., OTU_7 and *Loktanella* ssp., OTU_169), and an unclassified member of the family *Methylophilaceae* (OTU_15). Members from both bacterial families have been previously described to utilize some organic pollutants as a food source (Gutierrez et al., 2011; Chang et al., 2016). Other bacteria showed a negative impact from pollutant addition, which was neutralized when tDOM was added, likely due to the attachment of the toxic pollutants to tDOM and a consequent toxicity abatement. One important example is *Ilumatobacter* ssp. (OTU_29), which was one of the most abundant bacteria in the present study. The abundance of this OTU dropped by approximately 21% when only pollutants were added, while it did not significantly differ from the control when increased tDOM was also present. Other bacteria showed a positive synergistic effect from the combination of organic pollutants and tDOM additions. Examples of this trend are *Janthinobacterium* ssp. (OTU_1), *Paenibacillus* ssp. (OTU_79), *Yersinia* ssp. (OTU_144), and *Silvimonas* ssp. (OTU_343). These bacteria mainly originated from the soil addition and showed higher abundances when organic pollutants were also present. *Janthinobacterium* and *Paenibacillus* genera have been previously described to grow in water samples exposed to various organic pollutants (Hesselsoe et al., 2005; Grady et al., 2016). Finally, a negative combined effect from organic pollutants and tDOM additions was observed in some bacteria, preventing them to fully increase their growth under elevated tDOM conditions. Thus, Bacteria such as *Glacieola* ssp. (OTU_757), *Hahella* ssp. (OTU_1141), *Verrucomicrobia Subdivision3* (OTU_466), and *Bdellovibrio* ssp. (OTU_703) showed the highest abundances under increased tDOM conditions, but their growth was limited when organic pollutants were also present. Interestingly, this toxic effect was not observed when only pollutants (and not tDOM) were added. Some of these bacteria, such as *Glacieola* ssp., have been previously described to grow under elevated DOM concentrations (Sipler et al., 2017; von Scheibner et al., 2017). The toxicity effects observed on these bacteria could possibly be explained by the adsorption of organic pollutants onto tDOM (Ripszam et al., 2015a), increasing the direct contact and thus the toxicity.

The importance of the relative dose of some contaminants on bacterial diversity has been studied previously. For instance, cadmium has been described to have positive effects on bacterial diversity at intermediate concentrations, while negative effects were shown under high concentrations (Zhang et al., 2009). This points to a degree of tolerance of bacterial communities to certain levels of contaminants. Moreover, some contaminants may act as nutrient/energy source at suitable concentrations, only becoming toxic when their concentrations surpass toxicity threshold levels (Magalhães et al., 2011). In line with this, some pollutants added in this study could have been used as a nutrient/energy source, as may be indicated by bacteria responding positively to pollutant addition. In addition to the dose-dependent effects from pollutants (i.e., direct toxic effects), the importance of temporal exposure has also been defined as a relevant element for effects on the diversity of bacterial communities (Sun et al., 2012). Ager et al. (2010) reported negative effects of pentachlorophenol (organic pollutant also used in the present study) on bacterial diversity, and a subsequent recovery of community structure once the concentration of this pollutant had decreased after approximately 2 weeks. In contrast, in the present study we detected no obvious recovery over time, despite the decreased dissolved concentrations of many of the pollutants added; with stronger differences in community structure, diversity, and BP observed toward the end of the experiment. Although this may relate to the length of the experiment, this could be explained by the presence of multiple stressors (i.e., multiple types of organic pollutants), reducing the ability of some bacteria to develop tolerance to the harmful effects from multiple fronts (Vinebrooke et al., 2004). This aspect is an important consideration as environmental pollutants often occur in complex mixtures, and while the specific pollutant compounds and their concentrations may have easily definable impacts, the cumulative impacts can often be stronger.

It is worth noting that the addition of pollutants resulted in an additional increase in DOC concentration (Figure 1A), which was probably caused by two factors: the organic pollutants added contributing directly to the DOC pool (Ripszam et al., 2015b) and the use of methanol as the solvent in which the pollutant cocktail was solubilized for addition to the mesocosms. The clear increase in DOC concentration in pollutant treatments compared to their controls at week 1 decreased over the duration of the experiment, and this is very much in keeping with the decreasing trends in pollutants as they became associated and partitioned within the mesocosms (Ripszam et al., 2015b; Supplementary Figure S2). The concentration of methanol was not monitored during the experiment, and given that this compound is known to be used as carbon and energy source by a number of bacteria (Lidstrom, 1992), its presence could act as a confounding factor when discriminating between bacterial responses attributed to methanol or other organic pollutants. However, we found some strong significant correlations between specific pollutants and OTUs, suggesting a direct cause–effect relationship. One clear example in the present study is OTU_81, which corresponds to a betaproteobacterium belonging to the genus *Methylophilus*. It appears highly likely that the trends observed for this OTU were due to the utilization of methanol as a carbon and energy source, as described previously (Jenkins et al., 1987; Neufeld et al., 2008; Vishnivetskaya et al., 2010). This OTU was found to be positively affected by pollutant addition in both the presence and absence of increased tDOM concentration. Furthermore, the genus *Methylophilus* has been described to have an active role in the degradation of organic pollutants such as biphenyl (Jenkins et al., 1987; Uhlik et al., 2009) and anthracene (Zhang et al., 2011), among others, which were present in the cocktail of pollutants used in this study. In fact, biphenyl was one of the organic pollutants found to significantly correlate with *Methylophilus* abundance (Supplementary Table S6). All these correlations were negative, with an apparent delayed response of *Methylophilus* to the addition of pollutants (Figure 6), possibly due to the steady development of the population of this bacterial genus. While this bacterial genus barely represented 0.06% of the community when pollutants were not present, it became relatively abundant after long exposure to pollutants (up to 3.27% of the community by week 5).

### Bacteria as Possible Indicators of Environmental Disturbance

Identifying and tracking environmental disturbance is important and can inform on both direct human induced (e.g., pollutants) and climate change stressors. Since bacterial communities are capable of a rapid growth (doubling time up to 3 h in the present study) and can be readily influenced by changes to their environment, they could potentially represent interesting candidates as indicators of environmental disturbance. A number of OTUs that were relatively rare members of the natural bacterial assemblage became more abundant after the environmental disturbance induced by tDOM addition. Such proliferations of rare bacteria in response to environmental disturbance (e.g., tDOM, or pollutants) have been previously noted (Atlas et al., 1991; Campbell et al., 2011; Lindh et al., 2015). In our study, we also identified some OTUs which were only detected when tDOM was added, indicating that they were most likely bacteria specifically coming from the soil extract, and have the possibility to be incorporated into the marine community, as seen in previous studies (e.g., Traving et al., 2017). These bacteria were found in the soil extract (Supplementary Figure S4), and were recorded throughout the experiment in tDOM addition mesocosms. It should, however, be noted that the repeated addition of 1 μm-filtered fresh seawater (re-fill water) could have had a potential impact on the results, particularly the temporal development during the experiment. In the present study the technical constraints meant that some bacteria were regularly added to the system. Nevertheless, the additions of re-fill water to the mesocosm volume of 946 L of seawater resulted in a dilution factor of ∼50:1, and the fresh seawater was added to all mesocosms providing a normalizing effect across the treatments.

While further studies, for example functional and activity analyses, would be needed to confirm the specific role of certain taxa (e.g., survival, active role in the environment, contribution to net BP), it does appear that general microbial community analysis has the potential to offer some insights into environmental stressors. Furthermore, Sun et al. (2012) alluded to the utility of bacterial communities as rapid and sensitive indicators of environmental disturbance induced by the presence of contaminants. In this regard, we identified a number of OTUs significantly responding to the presence of organic pollutants alone and in combination with increased tDOM. Therefore, given the proliferation of specific bacterial taxa within the communities after this type of environmental disturbance, the rapid generation times displayed by bacterial species, their high sensitivity to disturbance induced by tDOM and organic pollutants, and the decreasing cost of metagenomics analyses, bacterial communities may represent rapid and efficient indicators to assess this type of environmental disturbance.

## Conclusion

To our knowledge, this is the first study on natural bacterioplankton communities under elevated tDOM concentration and in the presence of a mixture of organic pollutants. The addition of tDOM increased bacterial activity (i.e., BP and abundance) and also bacterial diversity; while the addition of organic pollutants led to an overall reduction of bacterial activity and diversity, particularly when comparing bacterial communities under elevated tDOM concentrations. We identified 32 OTUs contributing to the significant differences observed in community composition throughout the experiment, as well as 35 OTUs which responded differently to extended exposure to organic pollutants. These bacteria fell into six major trends according to their response to the stressors: (1) positive response to increased tDOM; (2) negative response to increased tDOM; (3) positive response to organic pollutant addition, which was removed by the addition of tDOM; (4) negative response to pollutant addition, which was neutralized when tDOM was added; (5) positive synergistic effect from the combination of organic pollutants and tDOM additions; 6) negative combined effect from organic pollutants and tDOM additions. Other minor trends included those bacteria that showed direct positive and negative responses to pollutant addition (including methanol). These findings indicate that the interaction between organic pollutants and tDOM, as well as their direct effects, can alter bacterial community structure and function. Furthermore, the changes catalyzed could conceivably have an impact on wider ecosystems processes such as deposition of pollutants to the benthos, microbial-mediated cycles of nutrients, carbon, and pollutants, and even the availability and transfer of pollutants in the food web.

## Author Contributions

ÅB, CG, AA, PH, MR, MT, and OR designed the mesocosm experiment. CG and MR carried out the contaminant analyses. OR, PH, and CG designed and conducted the microbial sampling. CG and DF carried out BP analyses. HS advised and supported molecular analyses and bioinformatics. ÅB conducted analyses of PP. JR, OR, and ST designed the molecular work. JR performed all molecular analyses, all bioinformatics analyses, and all statistical analyses. OR and ST provided support and supervision during manuscript drafting. JR conducted the manuscript writing and editing. All authors reviewed results and approved the final version of this manuscript.

## Conflict of Interest Statement

The authors declare that the research was conducted in the absence of any commercial or financial relationships that could be construed as a potential conflict of interest.

## Footnotes

1 https://www.ncbi.nlm.nih.gov/bioproject/
