# Corrigendum: Effects of Organic Pollutants on Bacterial Communities Under Future Climate Change Scenarios

**DOI:** 10.3389/fmicb.2019.02388

**Published:** 2019-10-18

**Authors:** Juanjo Rodríguez, Christine M. J. Gallampois, Sari Timonen, Agneta Andersson, Hanna Sinkko, Peter Haglund, Åsa M. M. Berglund, Matyas Ripszam, Daniela Figueroa, Mats Tysklind, Owen Rowe

**Affiliations:** ^1^Department of Microbiology, University of Helsinki, Helsinki, Finland; ^2^Department of Chemistry, Umeå University, Umeå, Sweden; ^3^Department of Ecology and Environmental Sciences, Umeå University, Umeå, Sweden; ^4^Umeå Marine Research Centre (UMF), Umeå University, Hörnefors, Sweden; ^5^Department of Equine and Small Animal Medicine, University of Helsinki, Helsinki, Finland; ^6^MSCi ApS Laboratory, Skovlunde, Denmark; ^7^Helsinki Commission (HELCOM), Baltic Marine Environment Protection Commission, Helsinki, Finland

**Keywords:** bacterial community composition, organic pollutants, dissolved organic matter, climate change, Baltic Sea, metagenomics

In the original article, there was a mistake in Figure 7 as published. The original version of Figure 7 was modified during the reviewing process, but was not properly uploaded; therefore, an older version was published instead. The original legend has not been changed. The corrected Figure 7 appears below.

**Figure 7:**
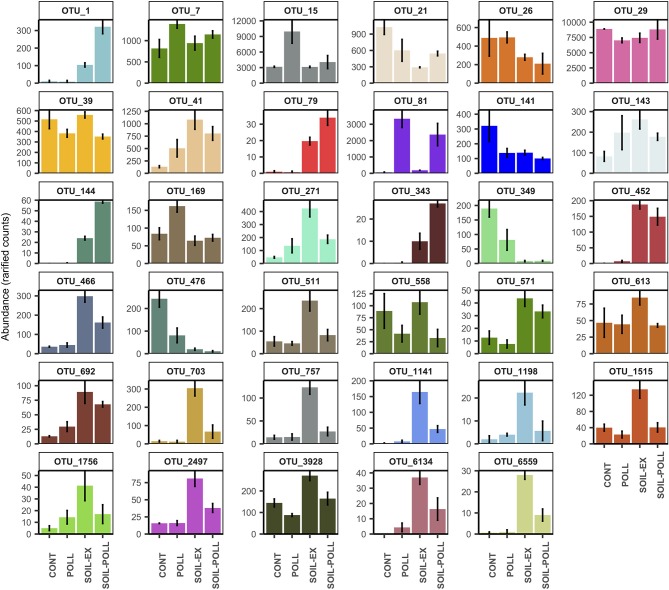
Abundance (rarified counts) of OTUs found at week 5 showing significant differences in abundance (one-way ANOVA) between polluted and non-polluted treatments (i.e., CONT vs. POLL and SOIL-EX vs. SOIL–POLL). Error bars represent the standard deviation (*n* = 3). Specific taxonomic classification corresponding to these OTUs is shown in Table 3.

The authors apologize for this error and state that this does not change the scientific conclusions of the article in any way. The original article has been updated.

