# Supplementary material for: Effects of Organic Pollutants on Bacterial Communities Under Future Climate Change Scenarios
Source: Front Microbiol. 2018 Nov 30;9:2926. doi: 10.3389/fmicb.2018.02926 (PMC6284067; doi:10.3389/fmicb.2018.02926)
Supplement: Supplementary file 1 [file Table_1.DOCX]

Table S1. DOC concentrations in the mesocosms from the different treatment and sampling times.
