# Supplementary material for: Effects of Organic Pollutants on Bacterial Communities Under Future Climate Change Scenarios
Source: Front Microbiol. 2018 Nov 30;9:2926. doi: 10.3389/fmicb.2018.02926 (PMC6284067; doi:10.3389/fmicb.2018.02926)
Supplement: Supplementary file 3 [file Table_3.DOCX]

Table S3. Likelihood ratio (LR) values from the generalized linear model (GLM) on bacterial classes found in the bacterial communities from the different treatments and over the experiment. The corresponding p-values are shown in Table 1. The GLM was applied in pairwise comparisons between the treatments. Bacterial classes are classified according to their relative abundance in the communities into abundant, moderate, and rare taxa. The factor “Sampling” refers to changes throughout the experimental time. LR values shown in bold correspond to p-values that were found to be significant (*p* < 0.05).

Adjusted p-values from the generalized linear model (GLM) on bacterial classes found in the bacterial communities from the different treatments. The GLM was applied in pairwise comparisons between the treatments. Bacterial classes are classified according to their relative abundance in the communities into abundant, moderate, and rare taxa. The factor “Sampling” refers to changes throughout the experimental time. Overall effects from the different pairwise comparisons are shown. Significant p-values are shown in bold.
