# Supplementary material for: Effects of Organic Pollutants on Bacterial Communities Under Future Climate Change Scenarios
Source: Front Microbiol. 2018 Nov 30;9:2926. doi: 10.3389/fmicb.2018.02926 (PMC6284067; doi:10.3389/fmicb.2018.02926)
Supplement: Supplementary file 4 [file Table_4.DOCX]

Table S4. Likelihood ratio (LR) values from OTUs that significantly contributed to the community composition differences observed between treatments (GLM analysis). LR values are shown for every pairwise comparison. The corresponding p-values are shown in Table 2. The taxonomic information provided is based on taxa names at class level and at the lowest level of classification (Silva v. 123) reached in every case. LR values shown in bold correspond to p-values that were found to be significant (p < 0.05).
