# Supplementary material for: Effects of Organic Pollutants on Bacterial Communities Under Future Climate Change Scenarios
Source: Front Microbiol. 2018 Nov 30;9:2926. doi: 10.3389/fmicb.2018.02926 (PMC6284067; doi:10.3389/fmicb.2018.02926)
Supplement: Supplementary file 5 [file Table_5.DOCX]

Table S5. Pearson’s correlation (r) between environmental factors and relevant OTUs from GLM test. Corresponding p-values are given below r values. For each OTU, class and taxon names are provided, the latter referring to the lowest taxonomic level successfully reached by SILVA database (i.e., genus, family, order, or class). Significant p-values are shown in bold. NaN stands for “Not a Number”, resulting from the no-occurrence of the OTUs in the corresponding pair-wise comparisons.
