# Supplementary figures and images for: Effects of Organic Pollutants on Bacterial Communities Under Future Climate Change Scenarios
Source: Front Microbiol. 2018 Nov 30;9:2926. doi: 10.3389/fmicb.2018.02926 (PMC6284067; doi:10.3389/fmicb.2018.02926)

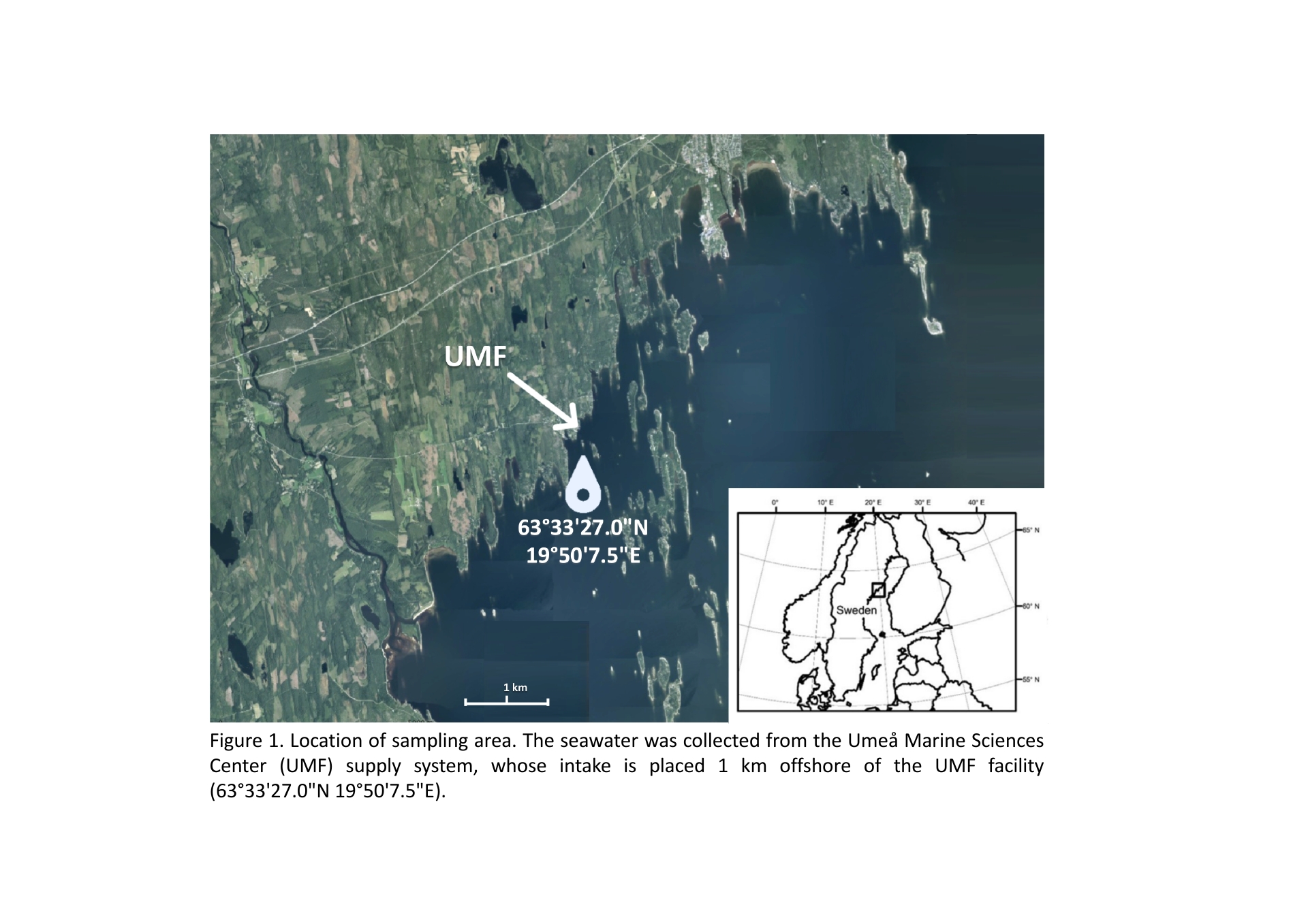

Supplement: Supplementary file 8 [file Image_1.JPEG]

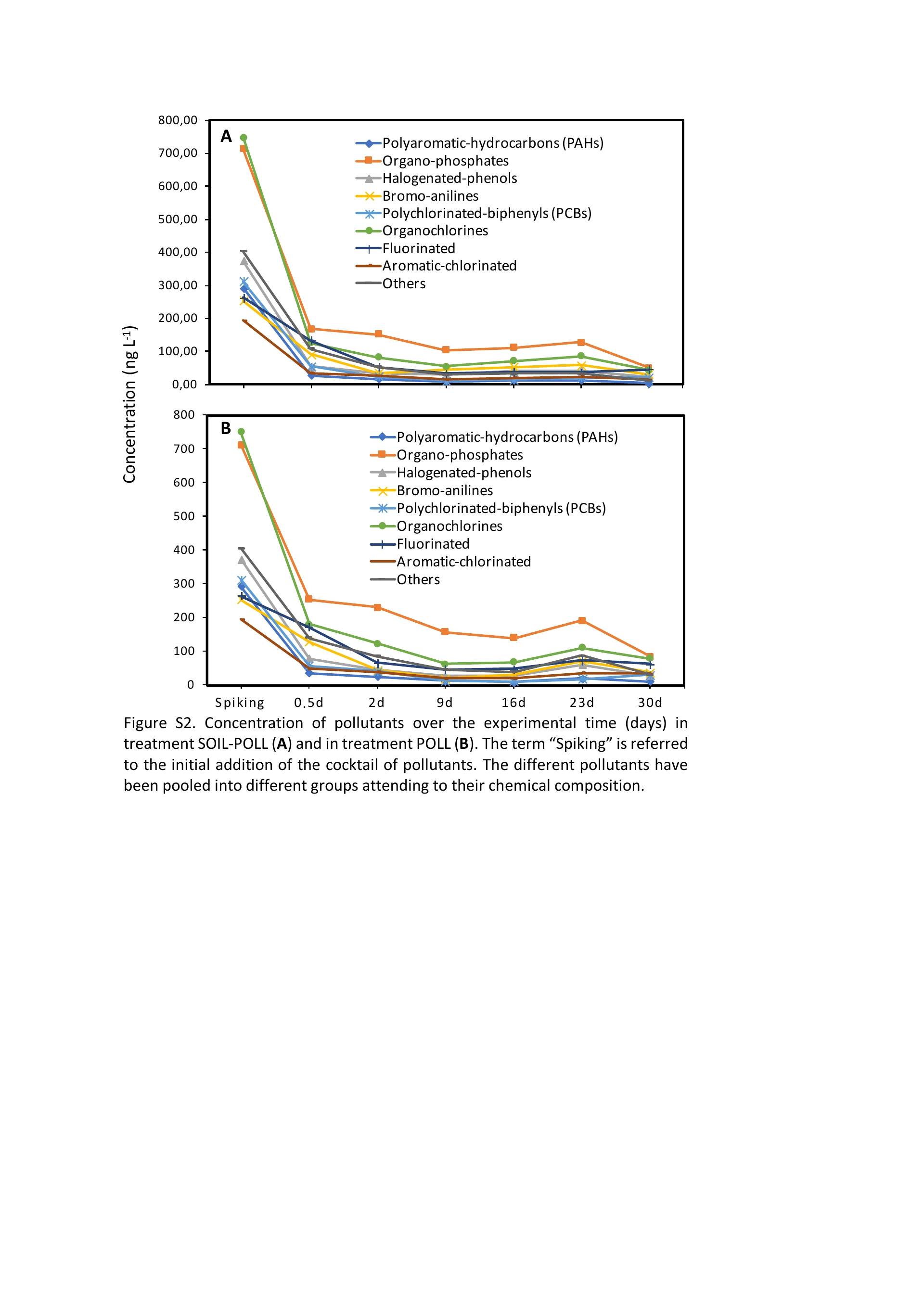

Supplement: Supplementary file 9 [file Image_2.JPEG]

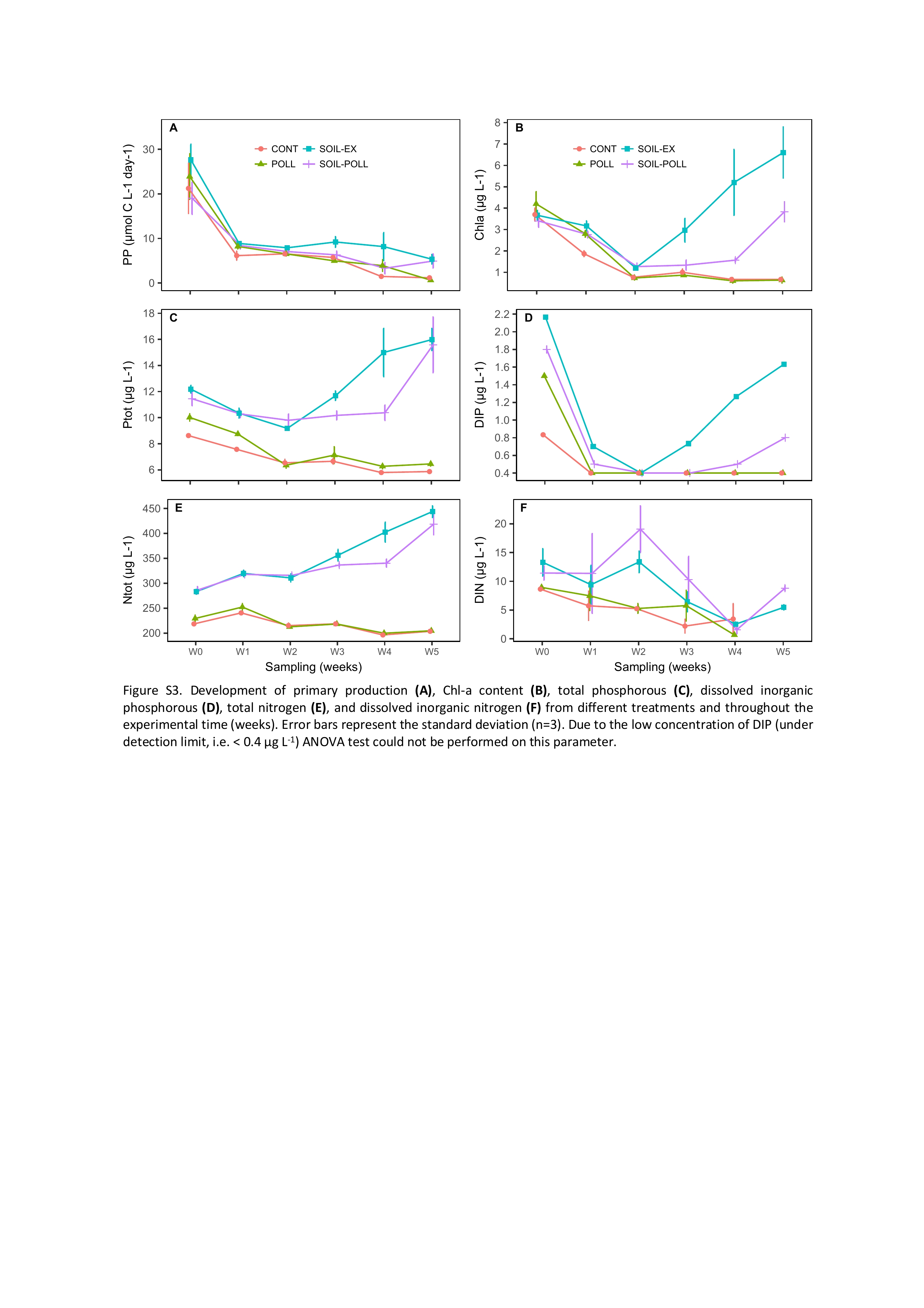

Supplement: Supplementary file 10 [file Image_3.JPEG]

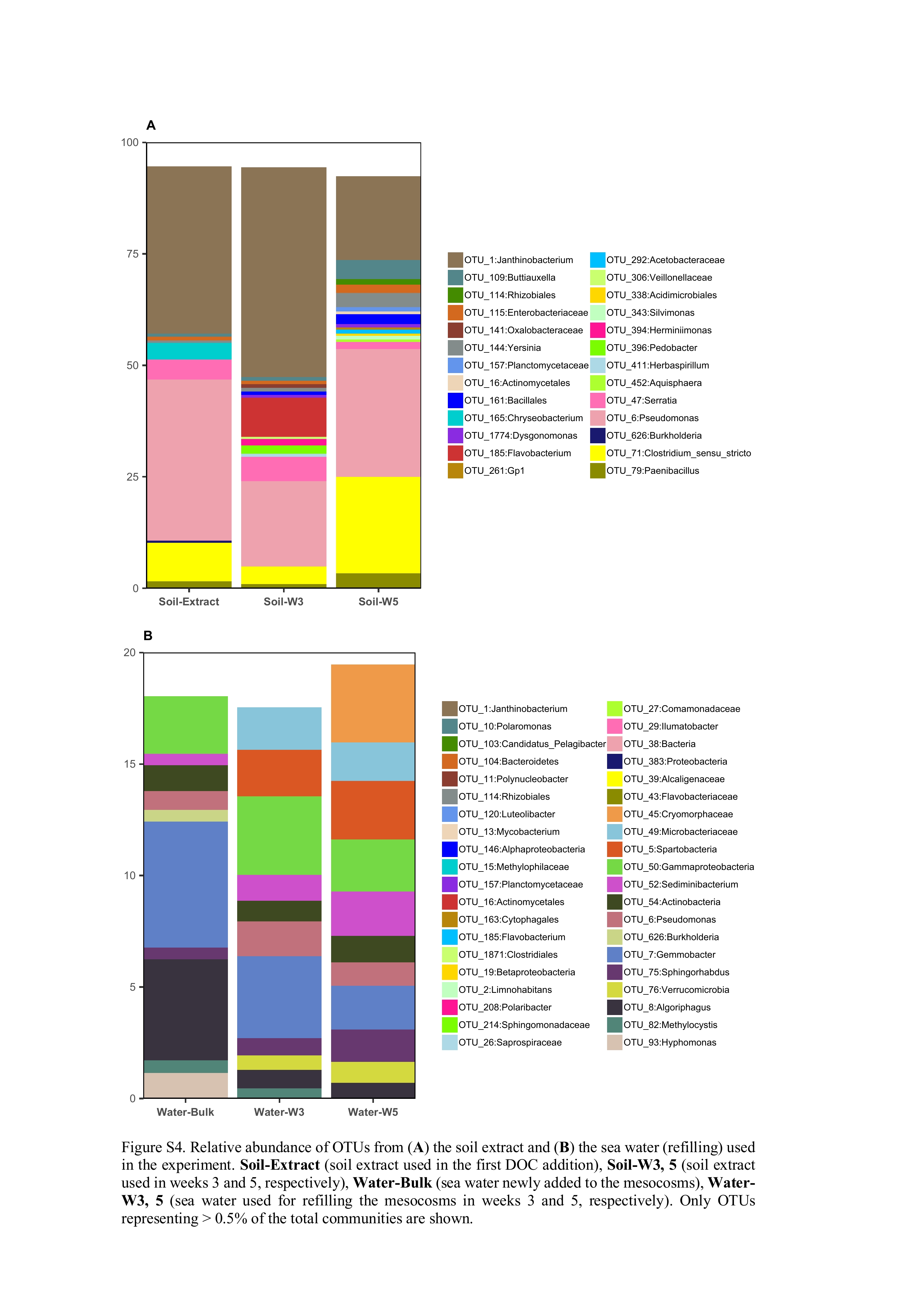

Supplement: Supplementary file 11 [file Image_4.JPEG]

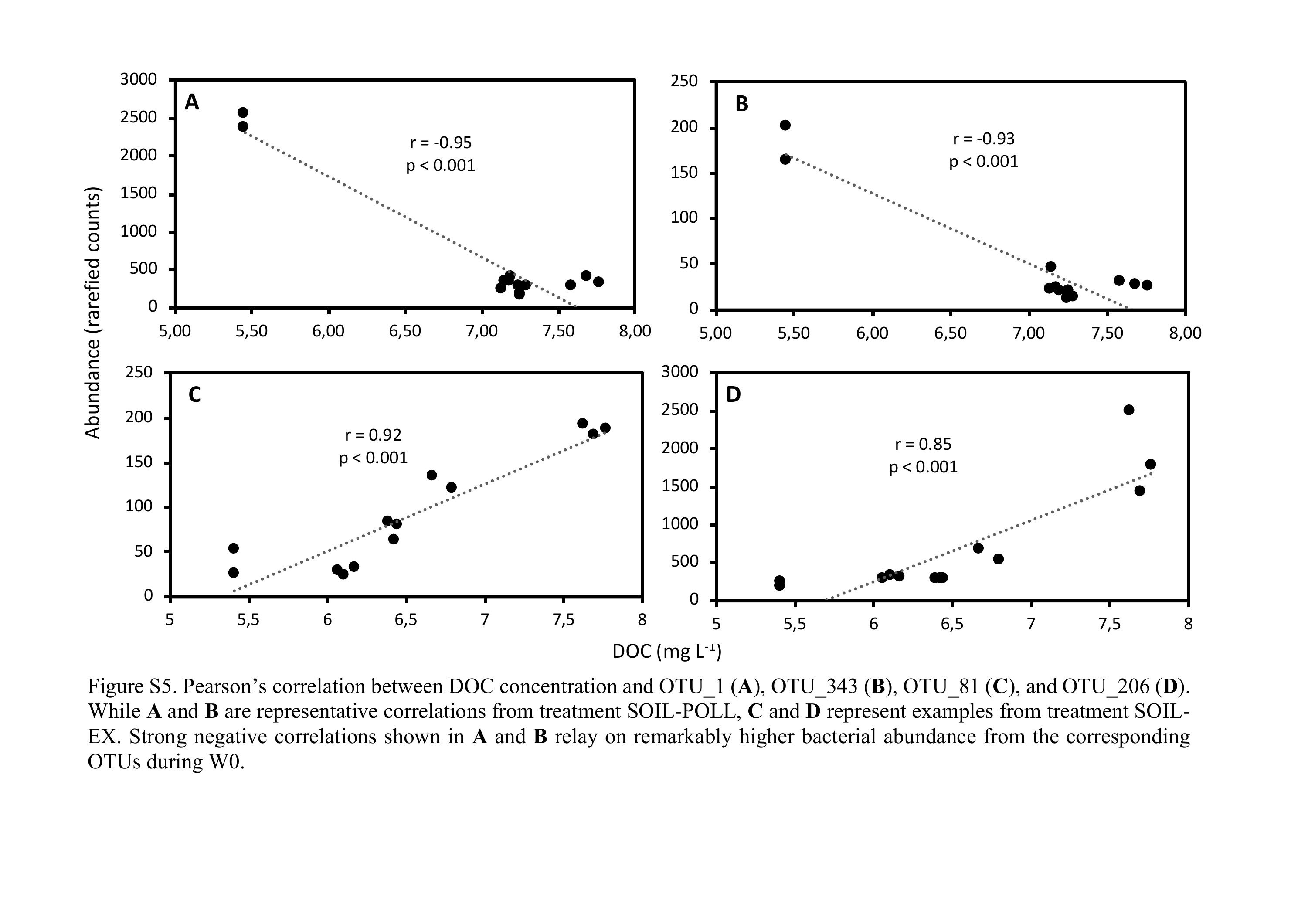

Supplement: Supplementary file 12 [file Image_5.JPEG]

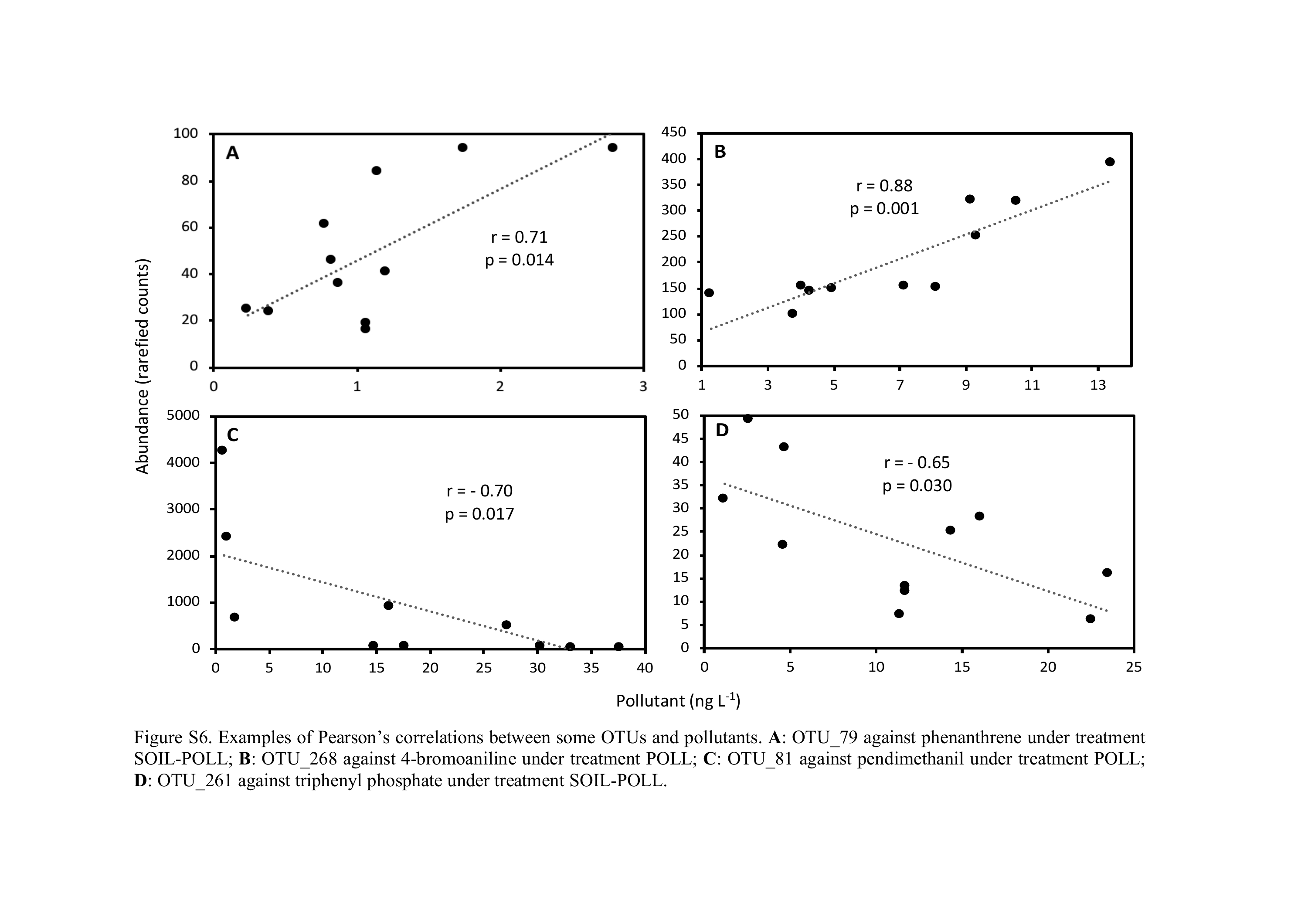

Supplement: Supplementary file 13 [file Image_6.JPEG]
